# Supplementary material for: Whole Genome Analysis of Ovarian Granulosa Cell Tumors Reveals Tumor Heterogeneity and a High-Grade TP53-Specific Subgroup
Source: Cancers (Basel). 2020 May 21;12(5):1308. doi: 10.3390/cancers12051308 (PMC7281495; doi:10.3390/cancers12051308)
Supplement: Supplementary file 1 [file cancers-12-01308-s001.zip › cancers-796571-suppl-figures.pdf]

Supplementary material: Whole Genome Analysis of Ovarian Granulosa Cell Tumors Reveals Tumor Heterogeneity and a High-Grade TP53-Specific Subgroup

Joline Roze, Glen Monroe, Joachim Kutzera, Jolijn Groeneweg, Ellen Stelloo, Sterre Paijens, Hans Nijman, Hannah van Meurs, Luc van Lonkhuijzen, Jurgen Piek, Christianne Lok, Geertruida Jonges, Petronella Witteveen, René Verheijen, Gijs van Haaften and Ronald Zweemer

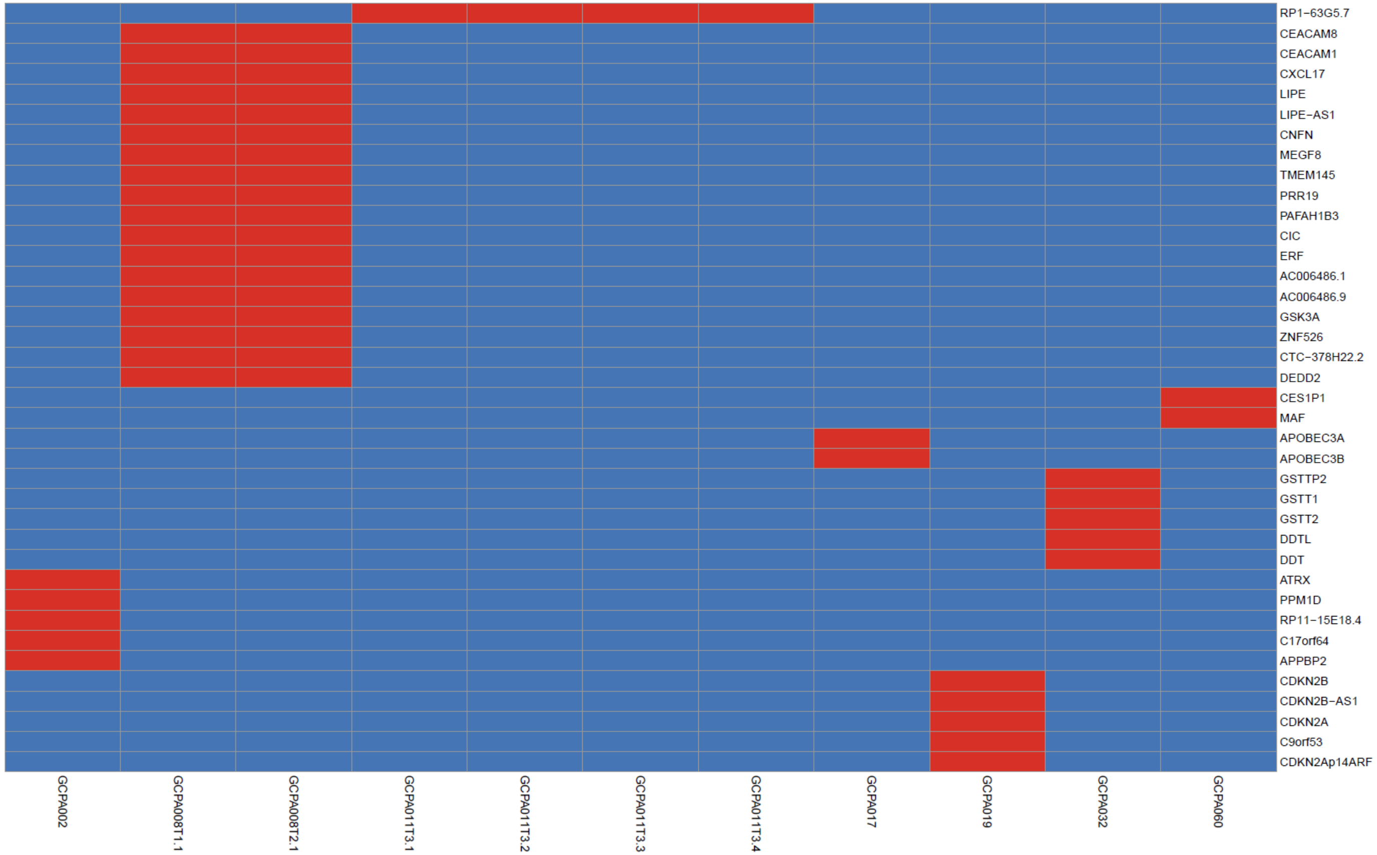

Figure S1. Full gene loss.

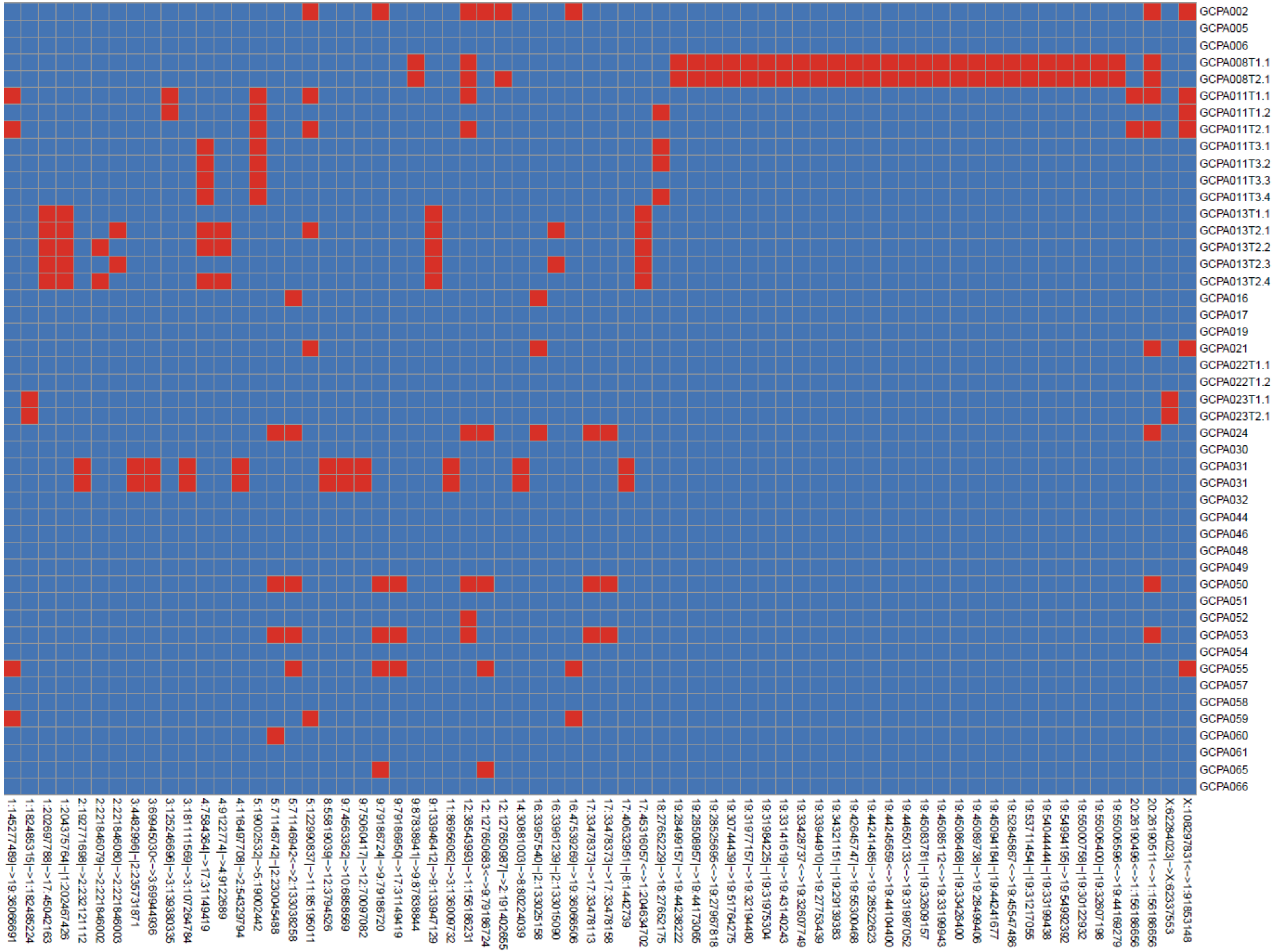
GCPA002
GCPA005
GCPA006
GCPA008T1.1
GCPA008T2.1
GCPA011T1.1
GCPA011T1.2
GCPA011T2.1
GCPA011T3.1
GCPA011T3.2
GCPA011T3.3
GCPA011T3.4
GCPA013T1.1
GCPA013T2.1
GCPA013T2.2
GCPA013T2.3
GCPA013T2.4
GCPA016
GCPA017
GCPA019
GCPA021
GCPA022T1.1
GCPA022T1.2
GCPA023T1.1
GCPA023T2.1
GCPA024
GCPA030
GCPA031
GCPA031
GCPA032
GCPA044
GCPA046
GCPA048
GCPA049
GCPA050
GCPA051
GCPA052
GCPA053
GCPA054
GCPA055
GCPA057
GCPA058
GCPA059
GCPA060
GCPA061
GCPA065
GCPA066

a

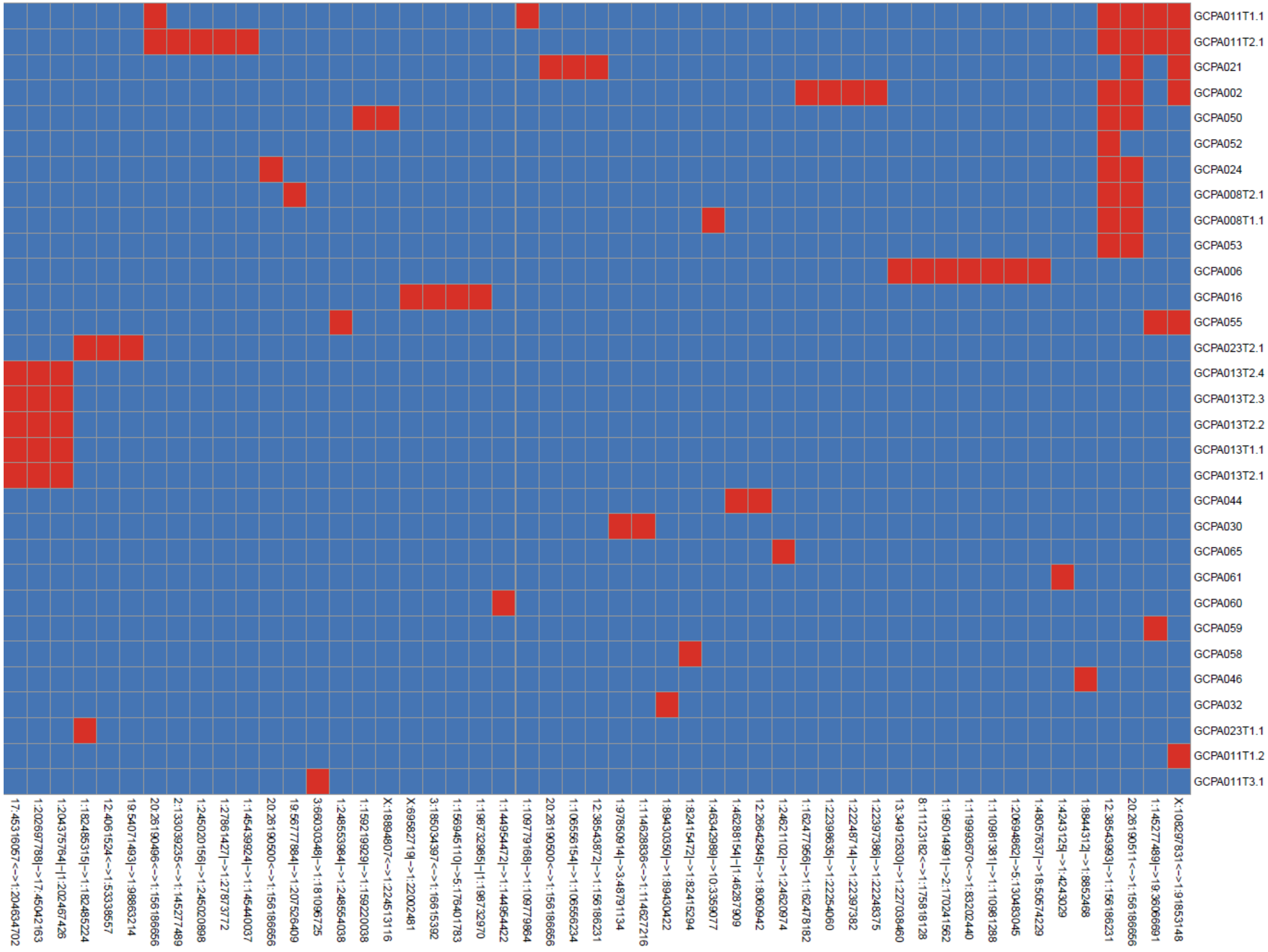
GCPA011T1.1
GCPA011T2.1
GCPA021
GCPA002
GCPA050
GCPA052
GCPA024
GCPA008T2.1
GCPA008T1.1
GCPA053
GCPA006
GCPA016
GCPA055
GCPA023T2.1
GCPA013T2.4
GCPA013T2.3
GCPA013T2.2
GCPA013T1.1
GCPA013T2.1
GCPA044
GCPA030
GCPA065
GCPA061
GCPA060
GCPA059
GCPA058
GCPA046
GCPA032
GCPA023T1.1
GCPA011T1.2
GCPA011T3.1
17:45316057<->1:204634702
1:202697788|->17:45042163
1:204375764|-|1:202467426
1:182485315|->1:182485224
12:4061524<->1:53338557
19:54071493|->1:98863214
20:26190496<->1:156186656
2:133039235<->1:145277489
1:245020156|->1:245020898
1:27861427|->1:27873772
1:145439924|->1:145440037
20:26190500<->1:156186656
19:56777884|->1:207526409
3:66030348|->1:181096725
1:248553984|->1:248554038
1:159219929|->1:159220038
X:1894807<->1:224513116
X:69582719|->1:22002481
3:185034397<->1:16615392
1:156945110|->5:176401783
1:198732985|-|1:198732970
1:144954472|->1:144954422
1:109779168|->1:109779864
20:26190500<->1:156186656
1:106556154|->1:106556234
12:38543872|->1:156186231
19:78509141|->3:48791134
1:114628836<->1:114627216
1:89430350|->1:89430422
1:82415472|->1:82415294
1:46342989|->10:3359077
1:46288154|-|1:46287909
12:26642845|->1:8060942
1:24621102|->1:24620974
1:162477956|->1:162478182
1:22398635|->1:22254060
1:22248714|->1:22397382
1:22397386|->1:22248375
13:34912630|->1:227033460
8:11123182<->1:175818128
1:195014991|->2:170241562
1:119993670<->1:83202440
1:110981381|->1:110981288
1:206948621|->5:130483045
1:48057637|->18:50574229
1:4243125|->1:4243029
1:8844312|->1:8852468
12:38543993|->1:156186231
20:26190511<->1:156186656
1:145277489|->19:36066691
X:108297831<->1:91853148
b

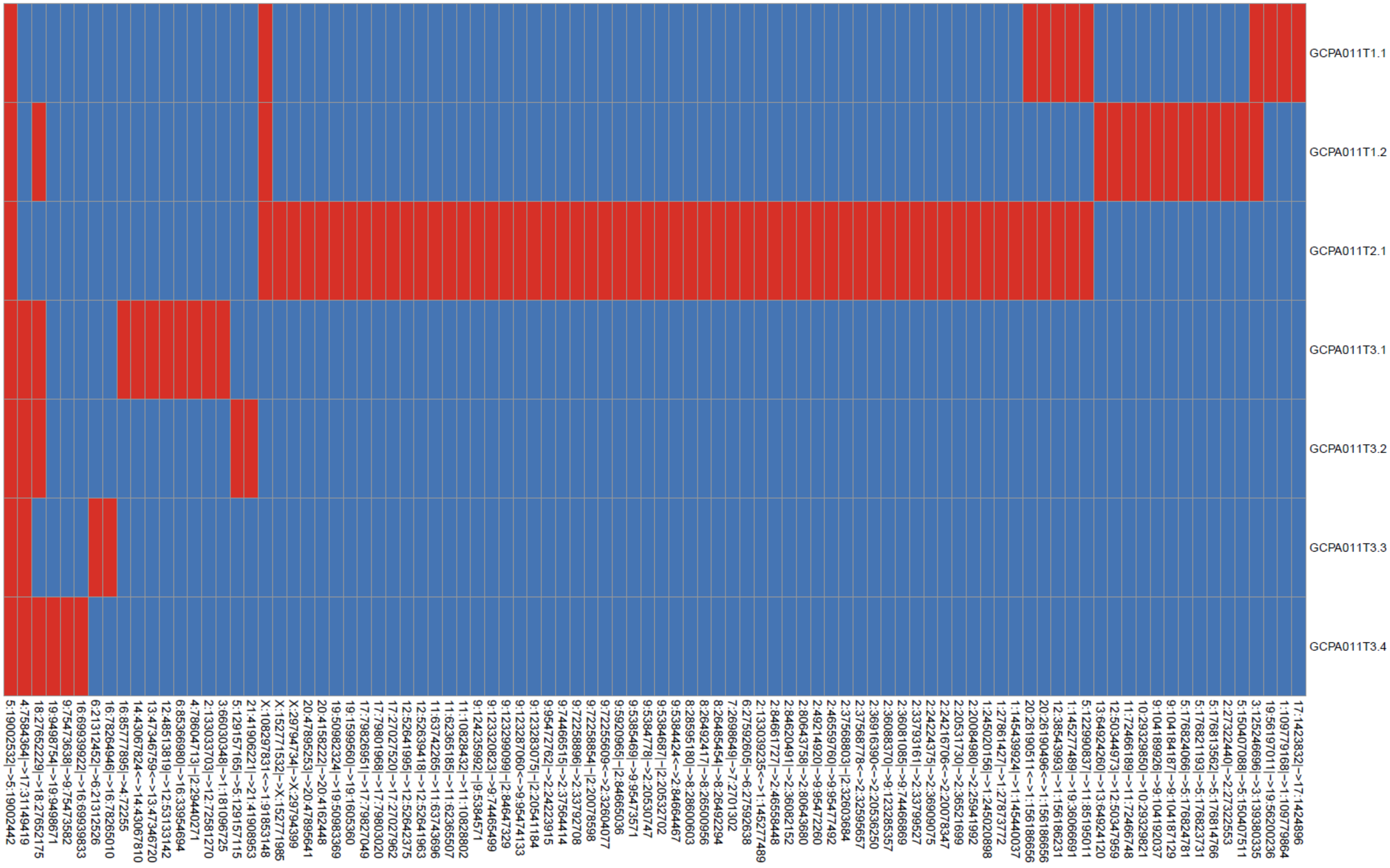
GCPA011T1.1
GCPA011T1.2
GCPA011T2.1
GCPA011T3.1
GCPA011T3.2
GCPA011T3.3
GCPA011T3.4

c

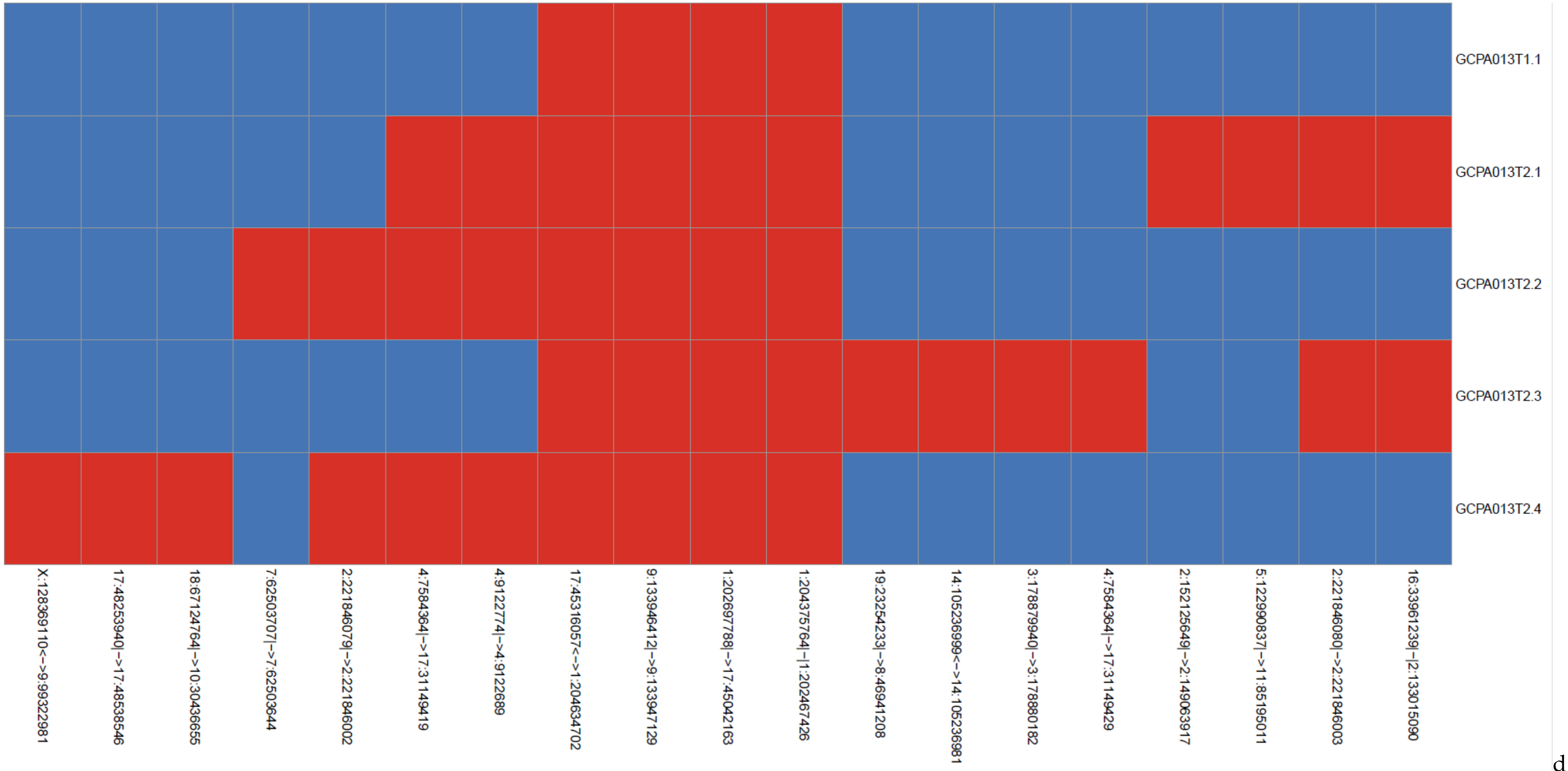

d

Figure S2. Structural Variants. **a**. Structural Variants in more than 1 sample**. b**. Structural Variants in chromosome 1***. c**. Structural Variants in patient 11**. d**. Structural Variants in patient 13**.** In the labels, "|->" marks a front-to-back breakpoint (preserved direction), "|-|" a back-to-back breakpoint and "<->" a front-to front breakpoint. *The overlapping breakpoint shown in the figure are not exactly on the same position, but detected within a range of 50 base pairs.

GCPA002

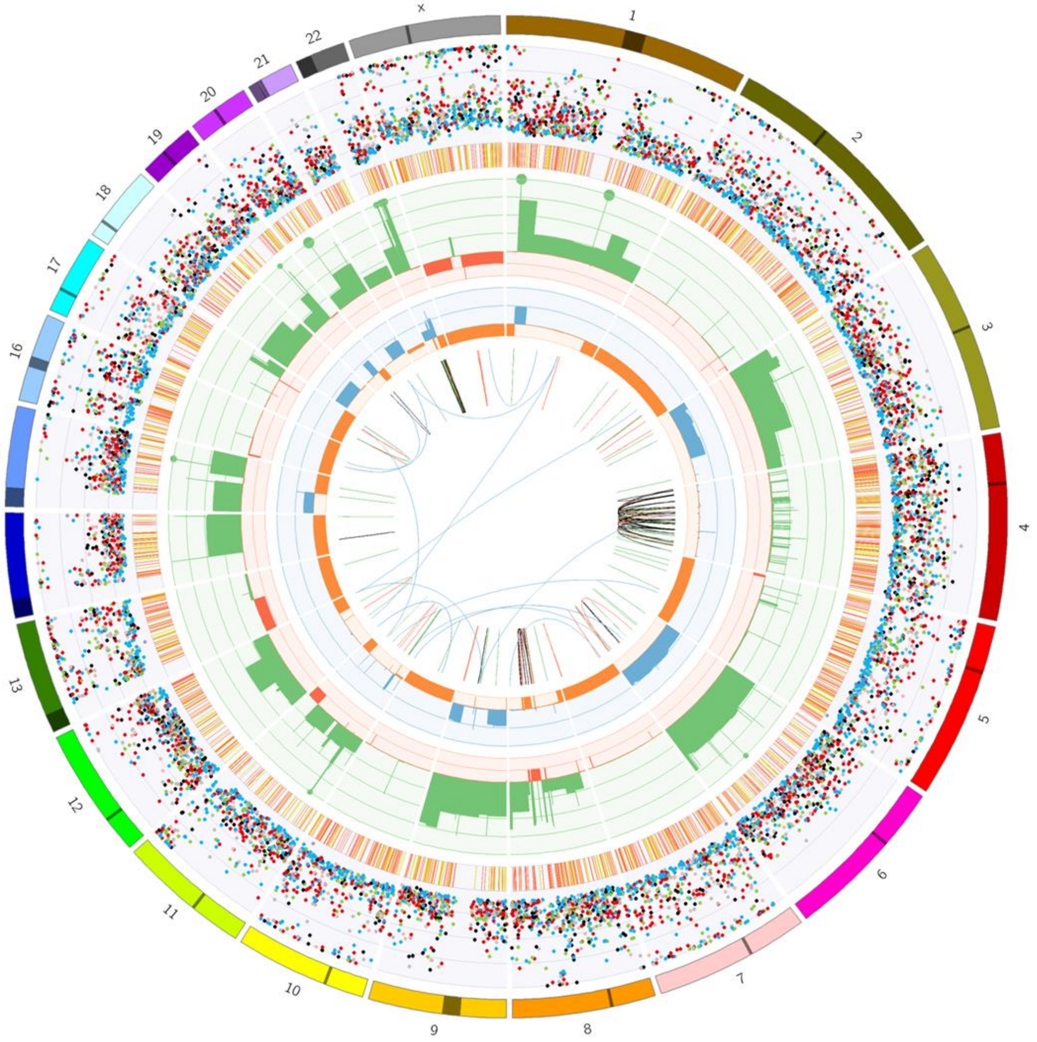

GCPA016

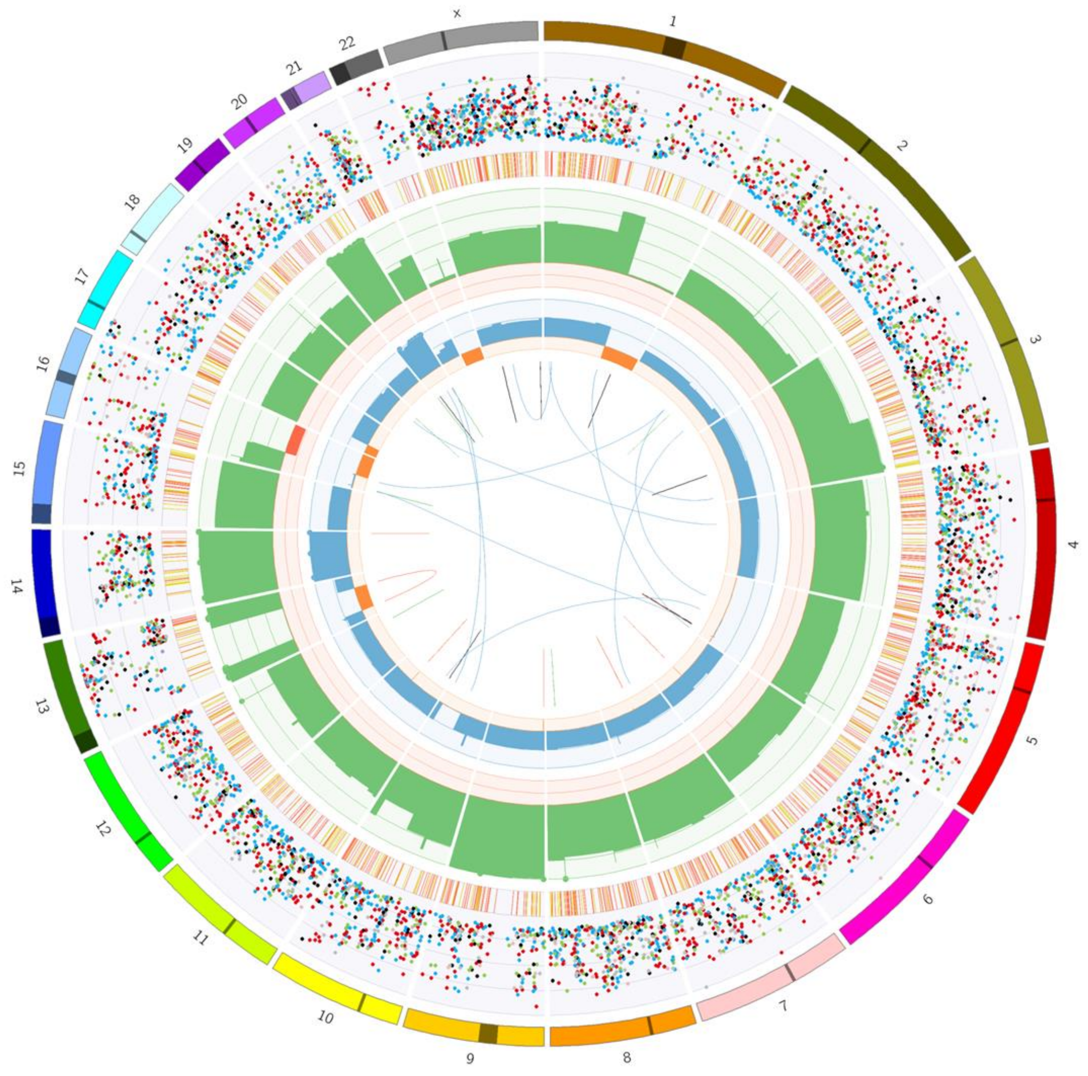

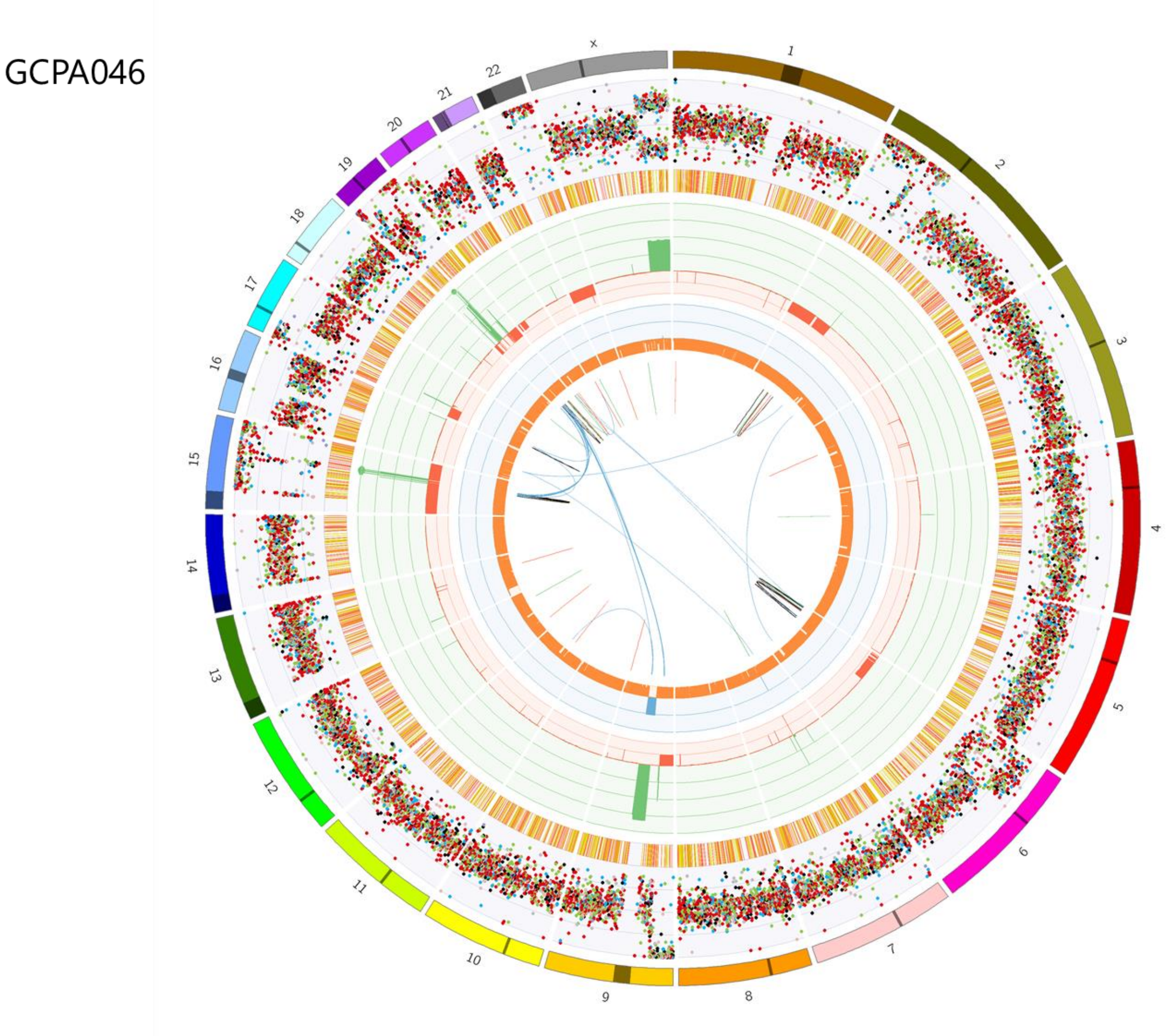

Figure S3. Circos plots and venn diagrams. a. Circos plots of TP53 mutant samples.

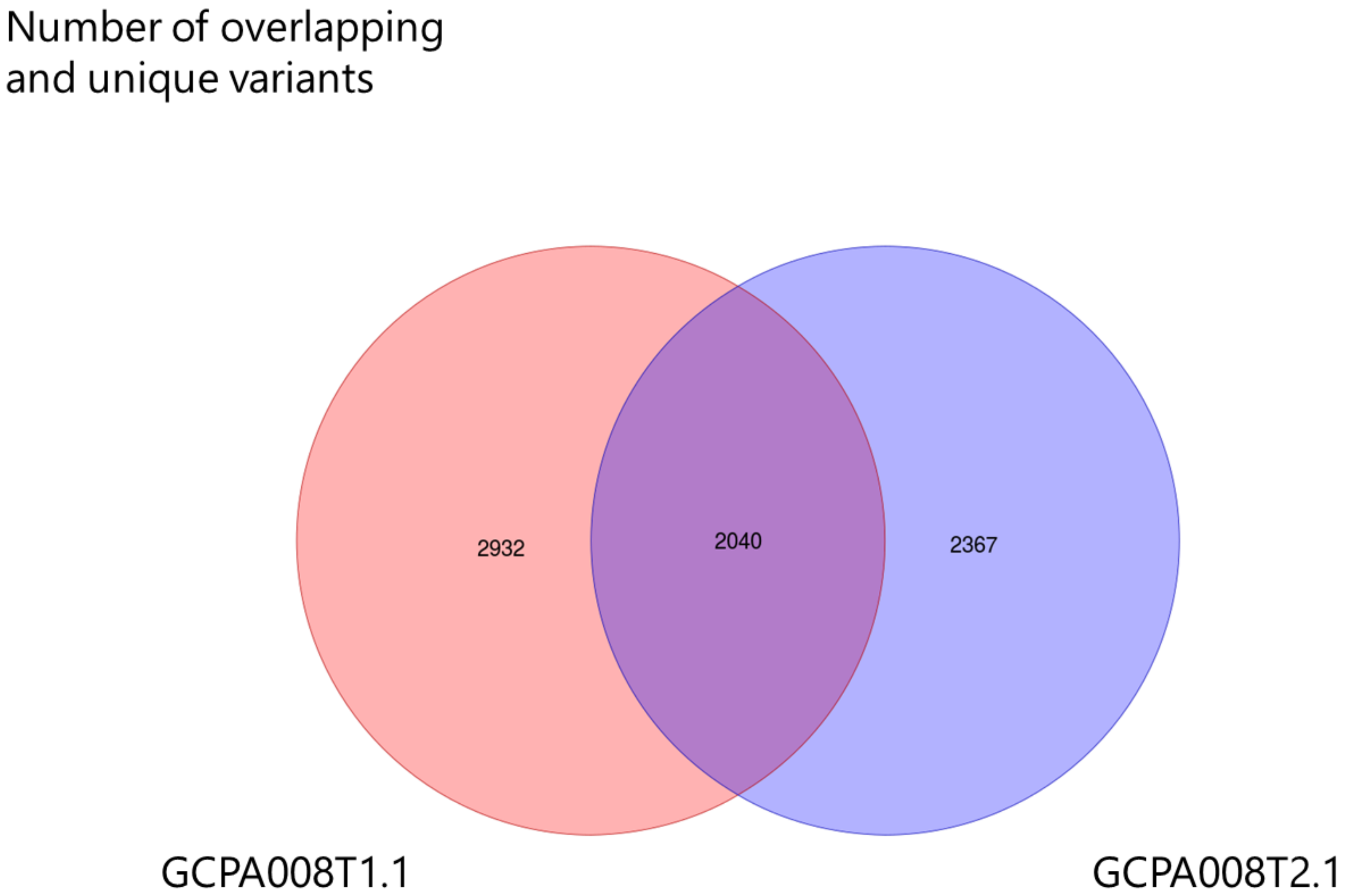
Number of overlapping
and unique variants
2932
2040
2367
GCPA008T1.1
GCPA008T2.1

GCPA008T1.1

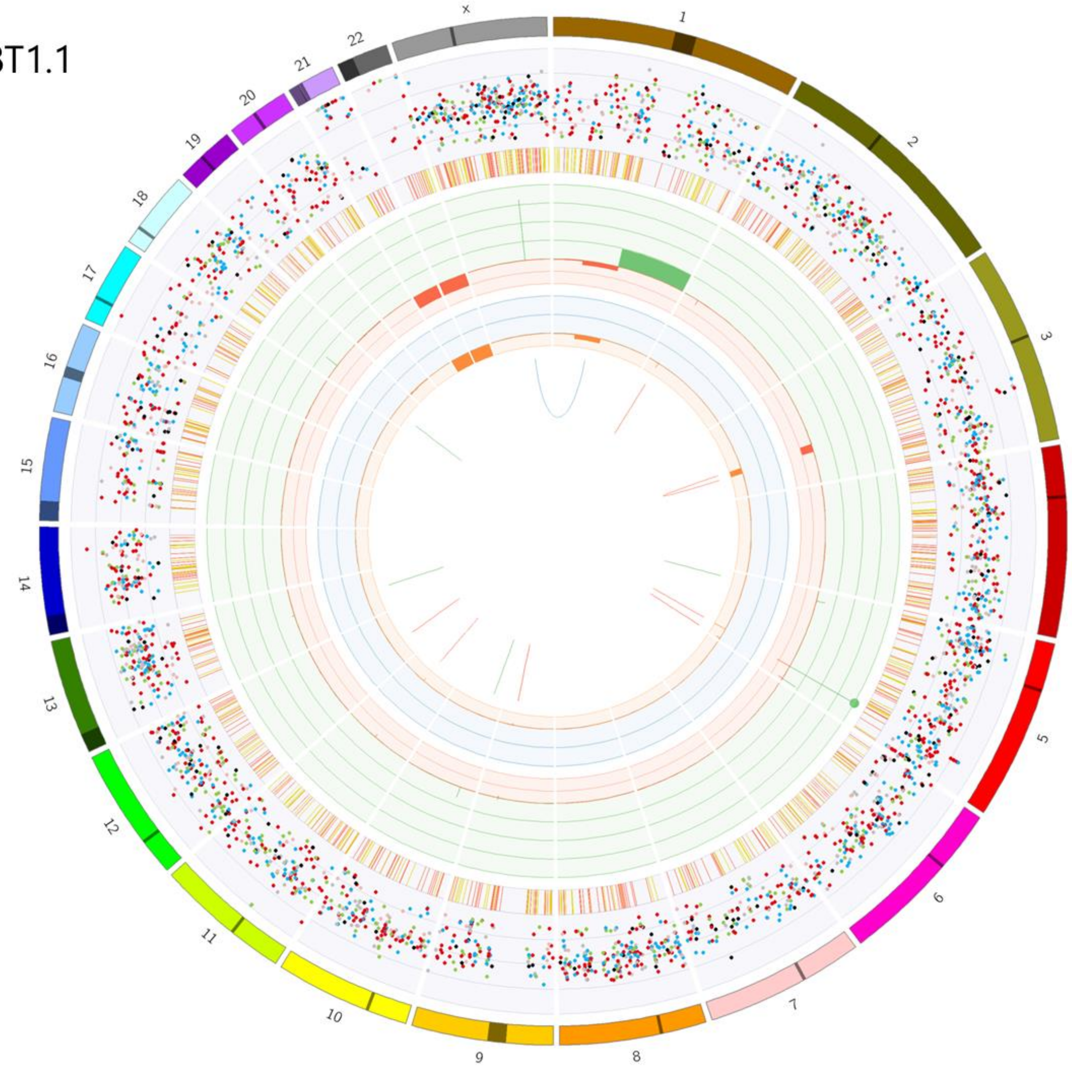

GCPA008T2.1

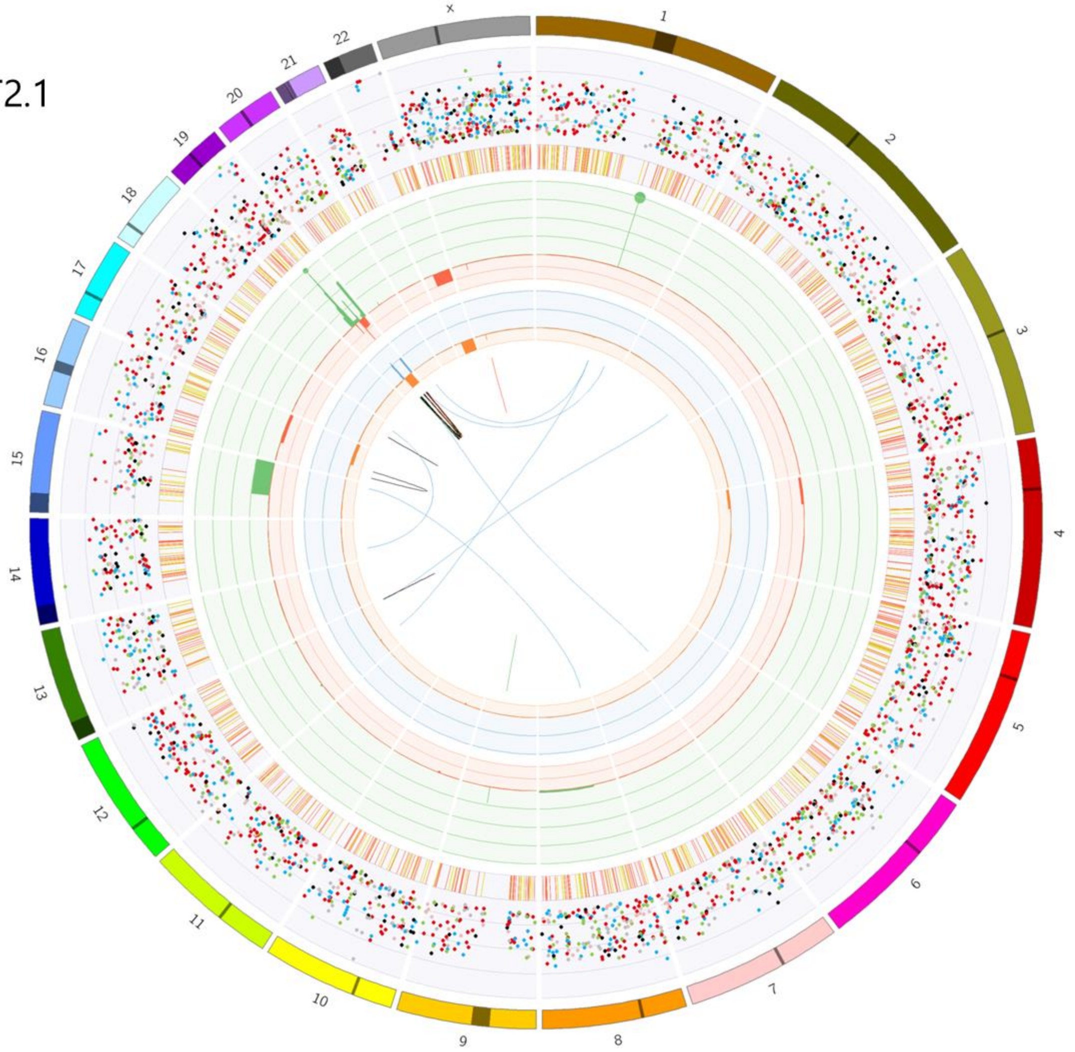

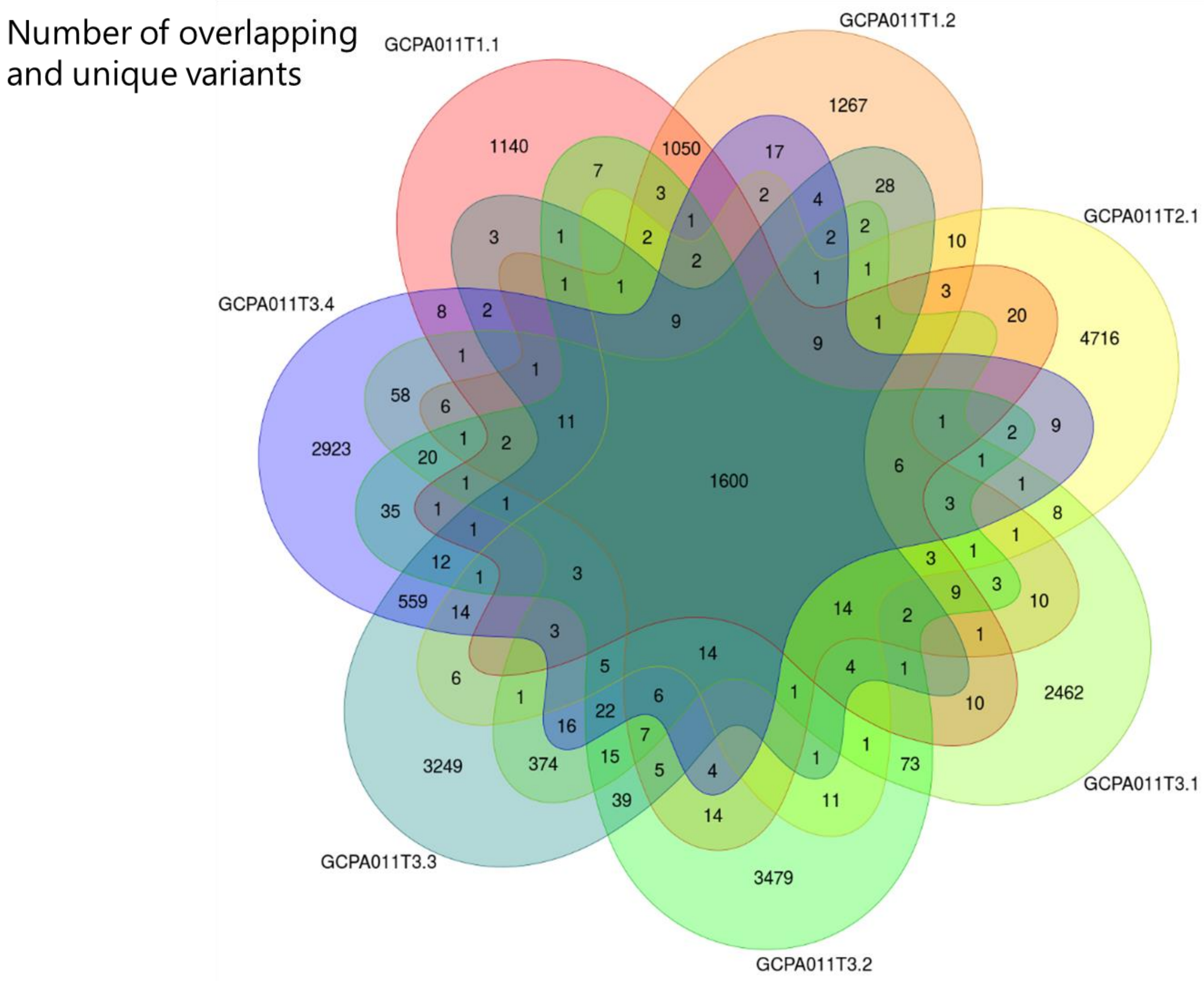
Number of overlapping and unique variants
GCPA011T1.1
GCPA011T1.2
GCPA011T2.1
GCPA011T3.4
GCPA011T3.1
GCPA011T3.3
GCPA011T3.2

GCPA011T1.1

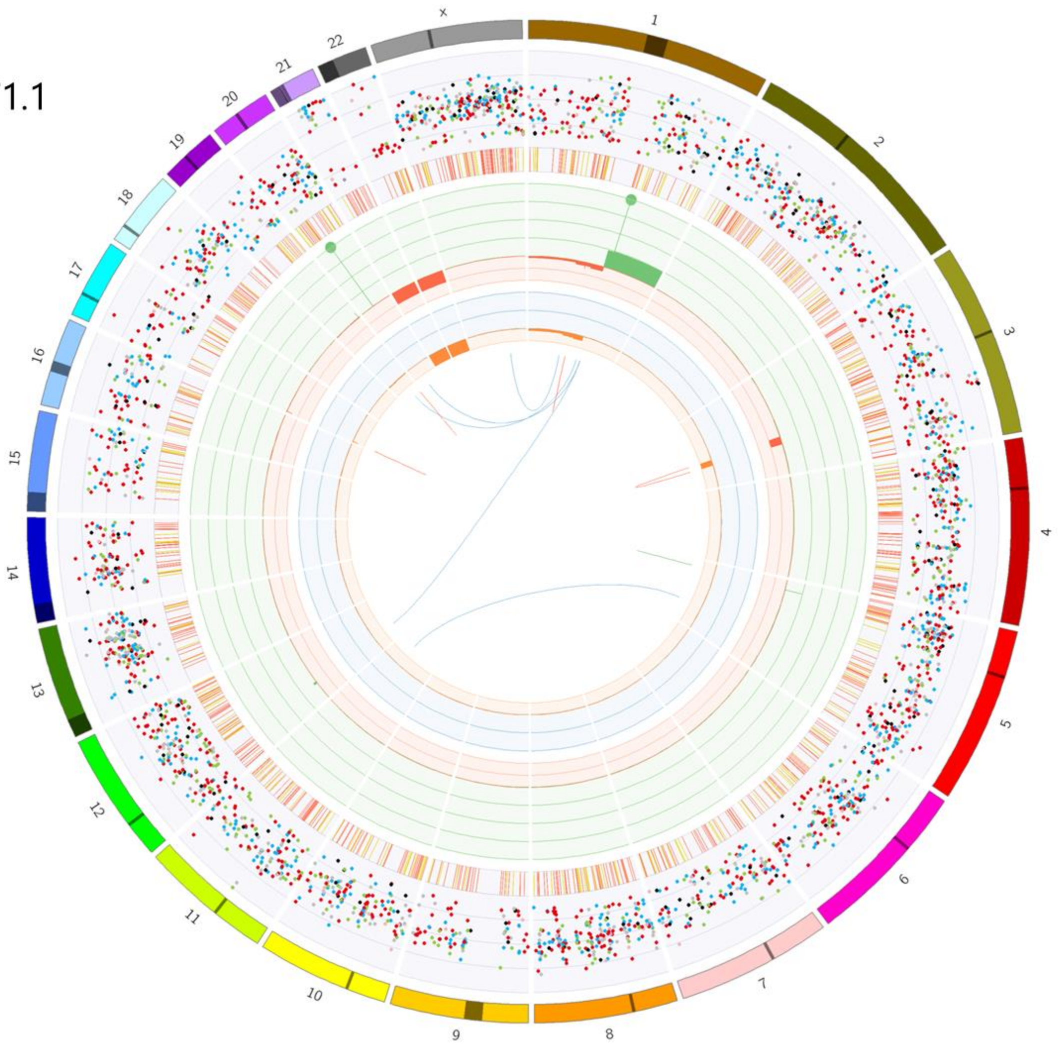

GCPA011T1.2

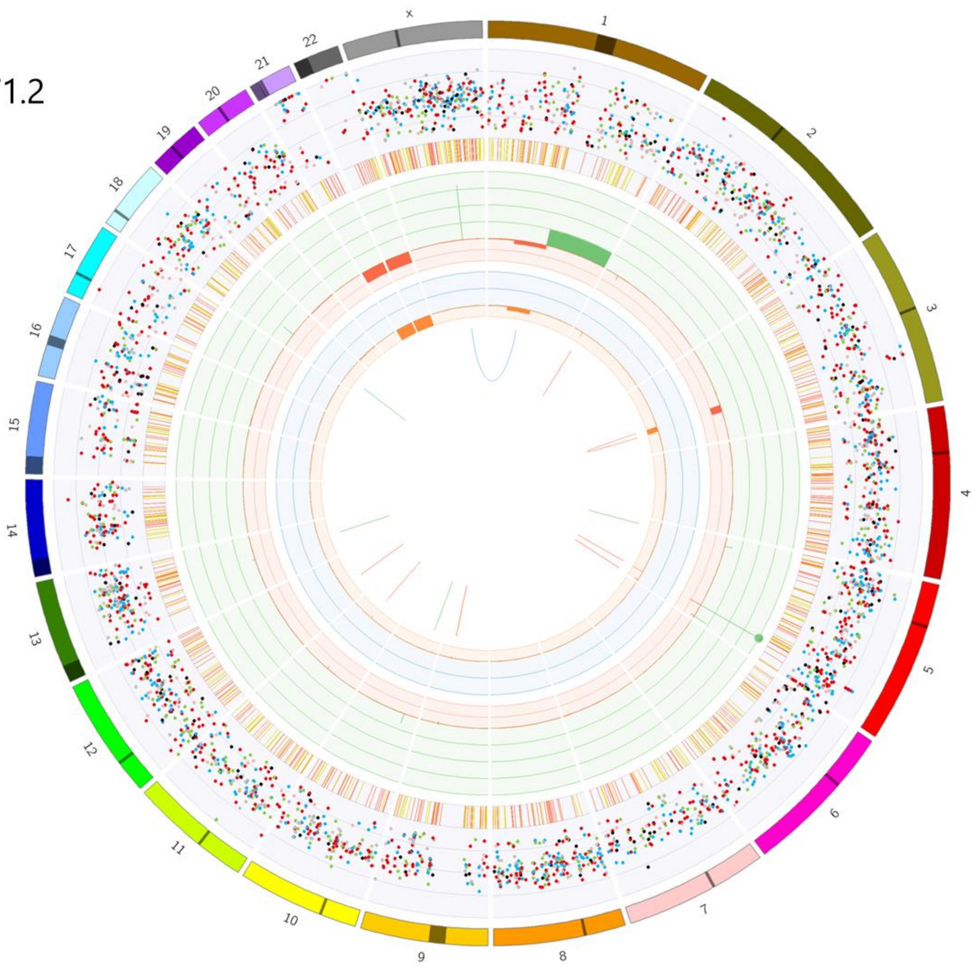

GCPA011T2.1

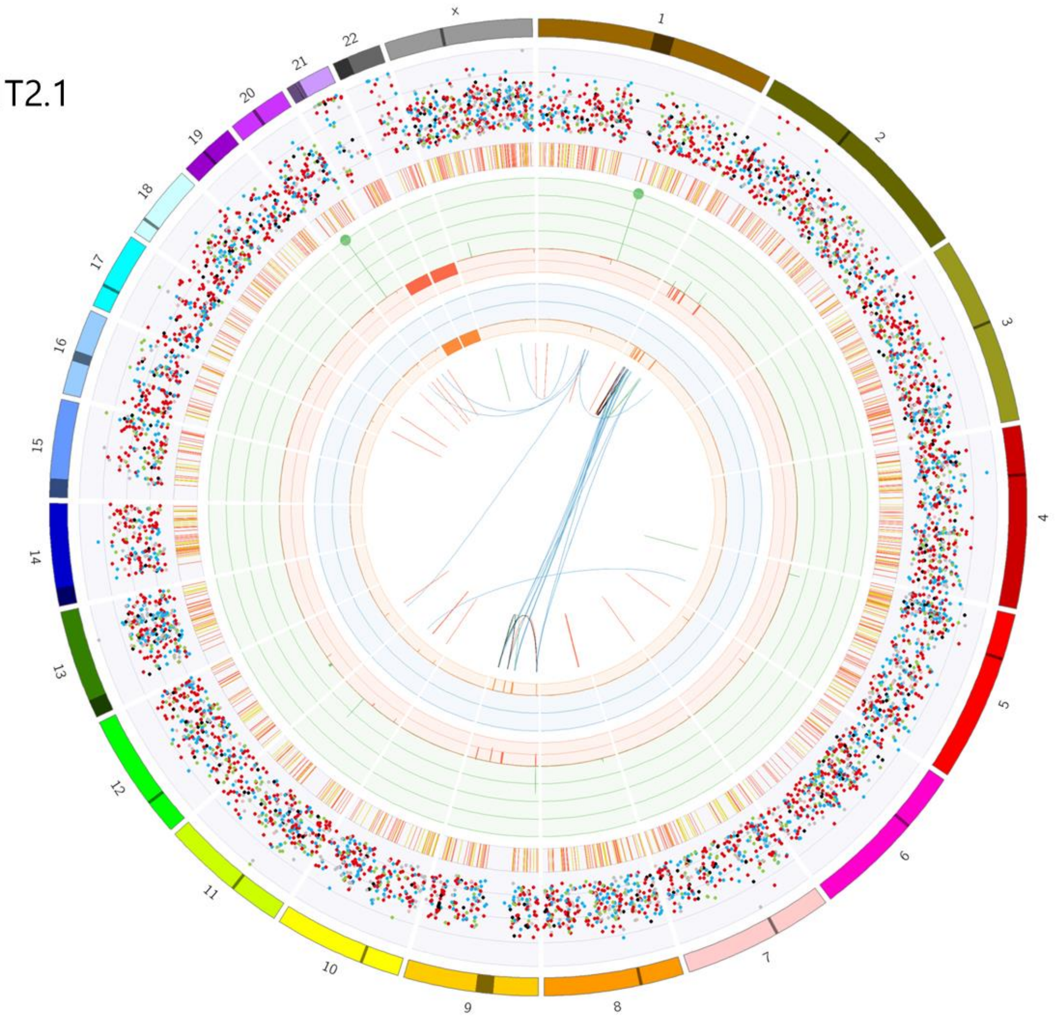

GCPA011T3.1

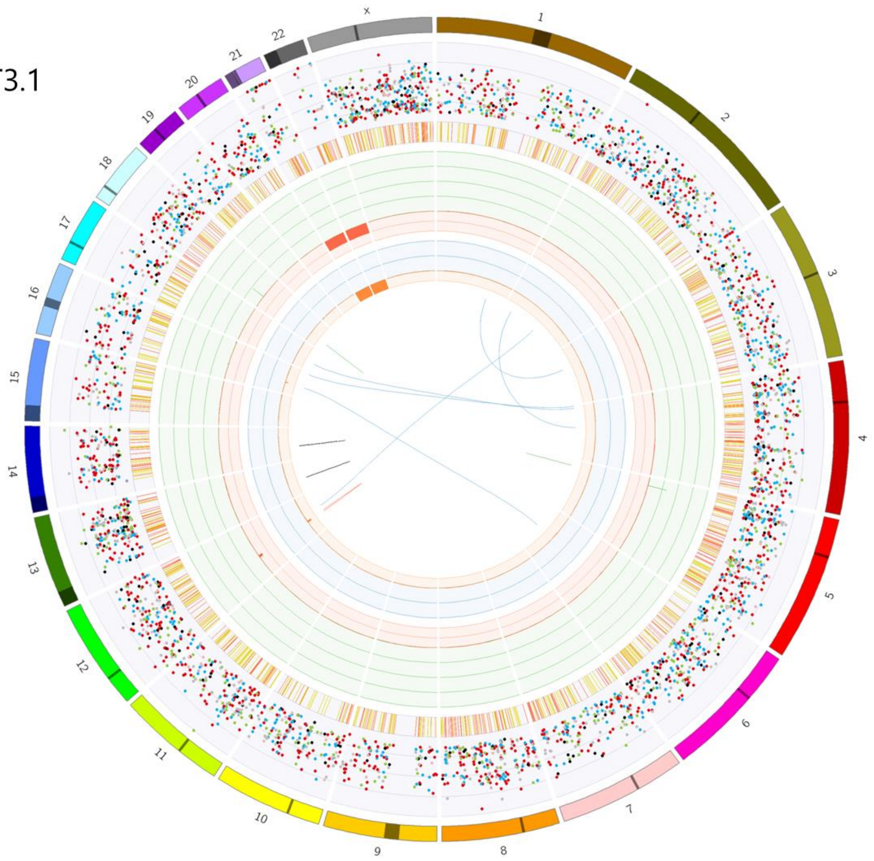

GCPA011T3.2

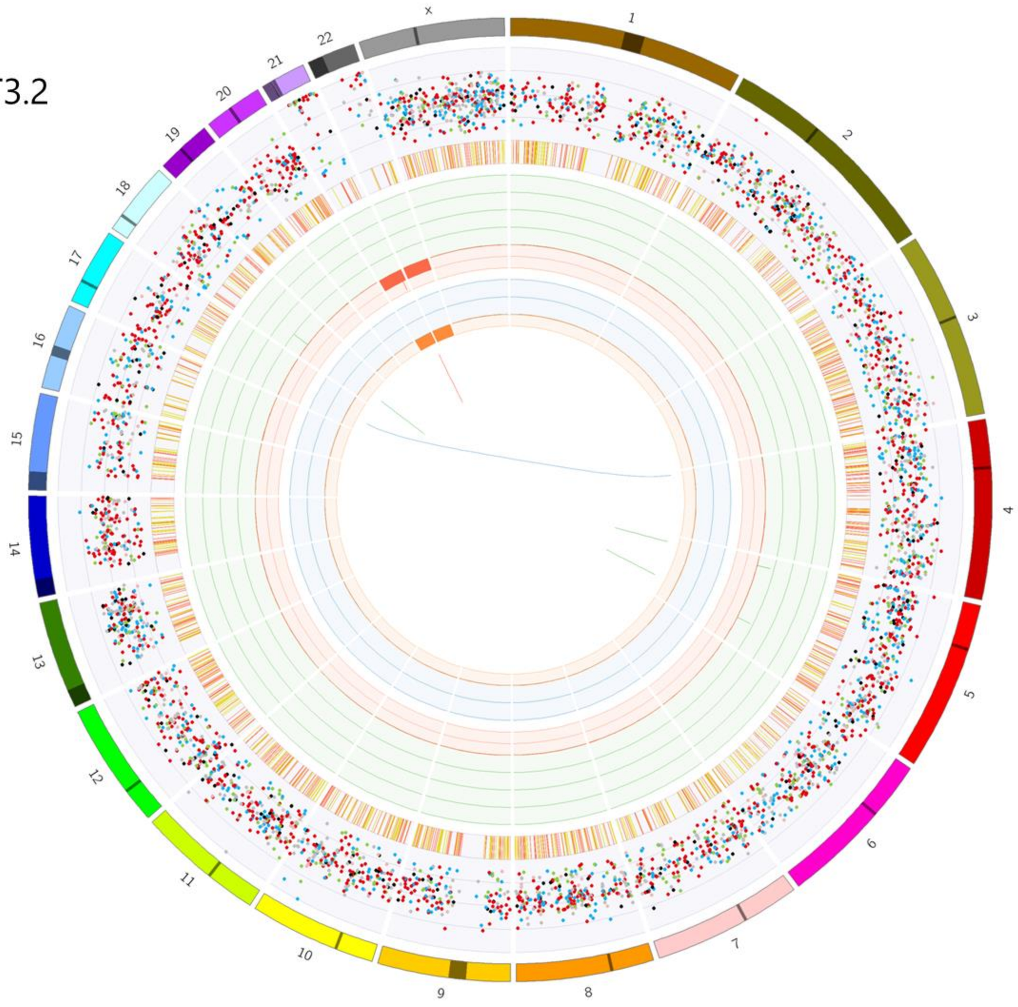

GCPA011T3.3

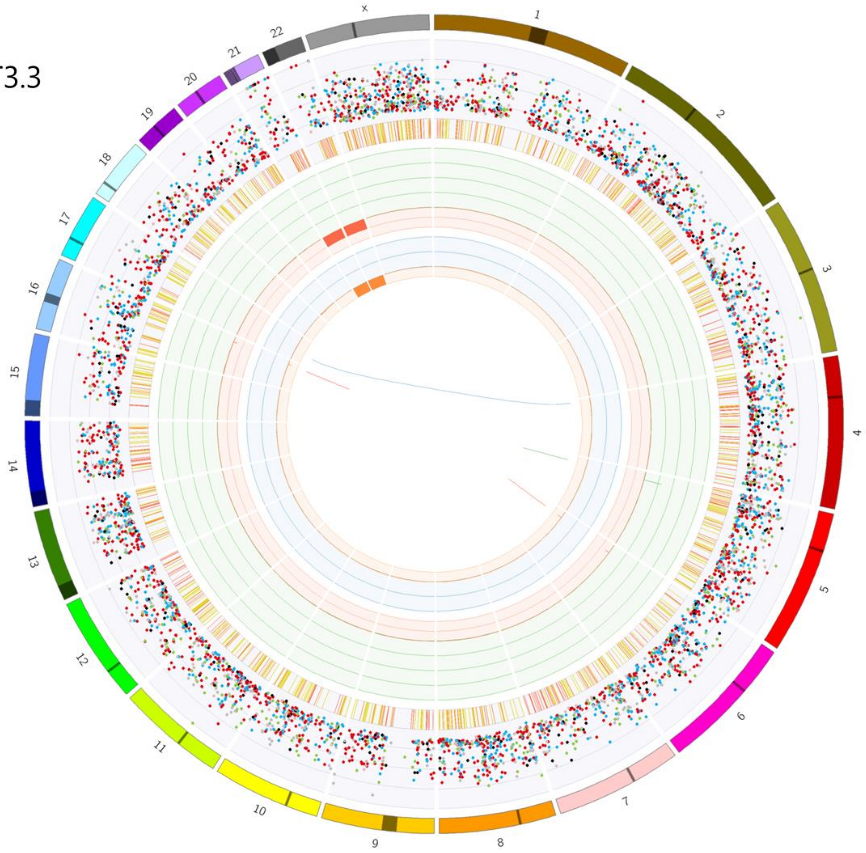

GCPA011T3.4

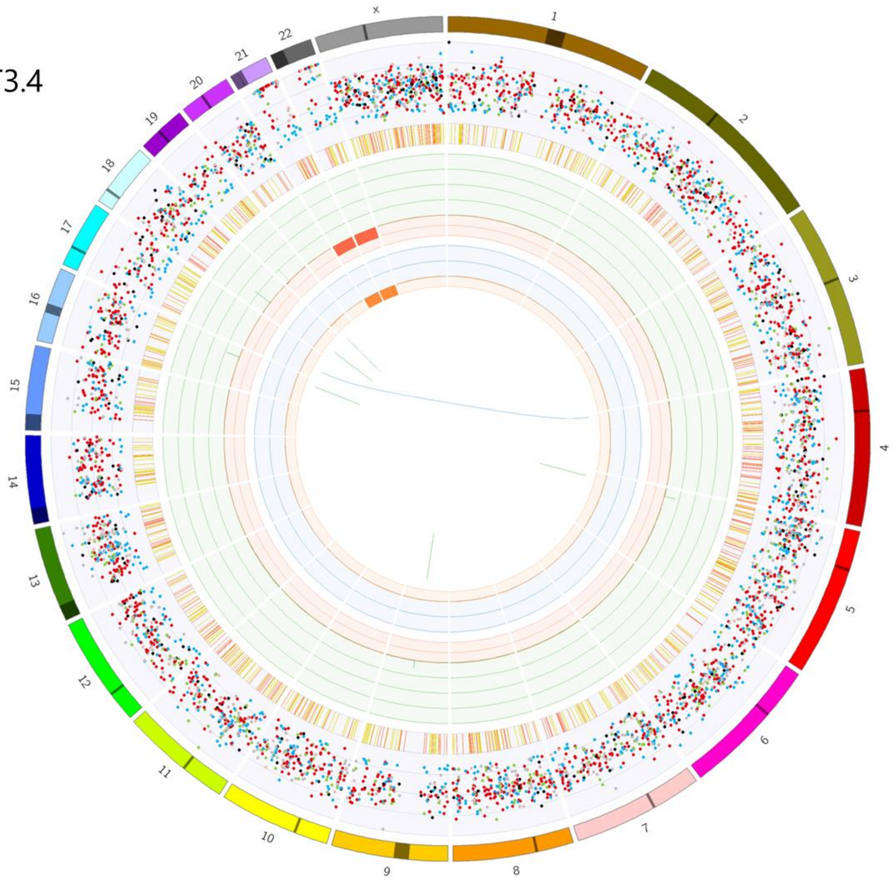

Number of overlapping and unique variants

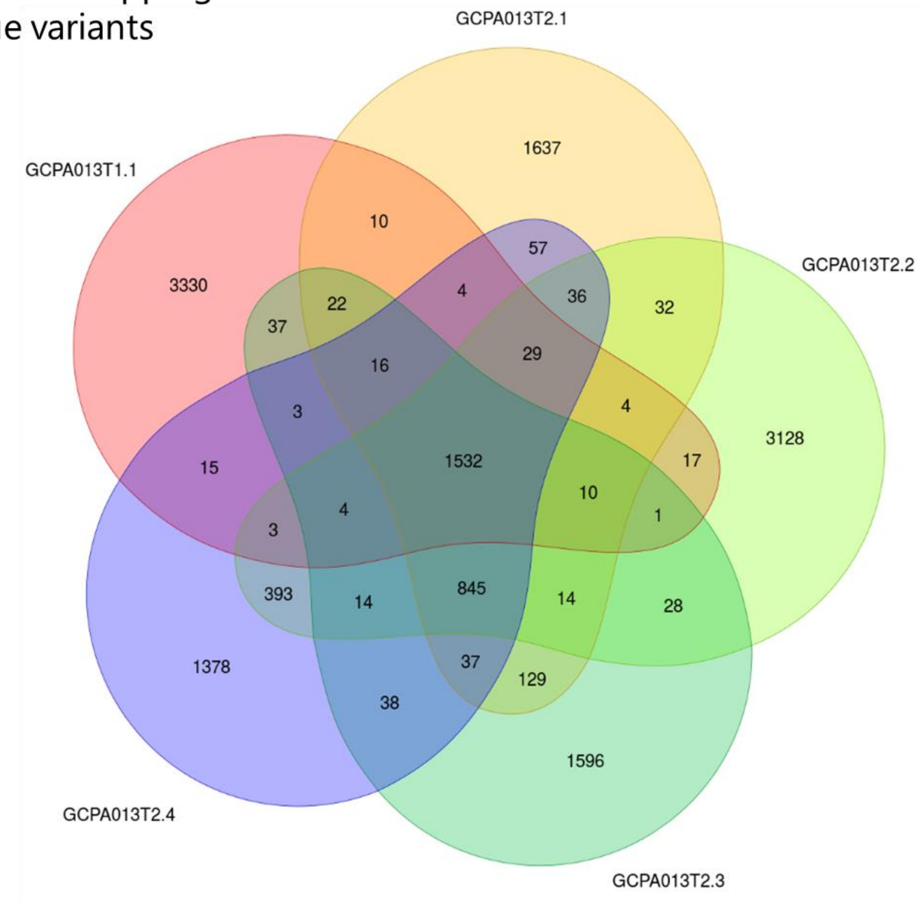

GCPA013T1.1

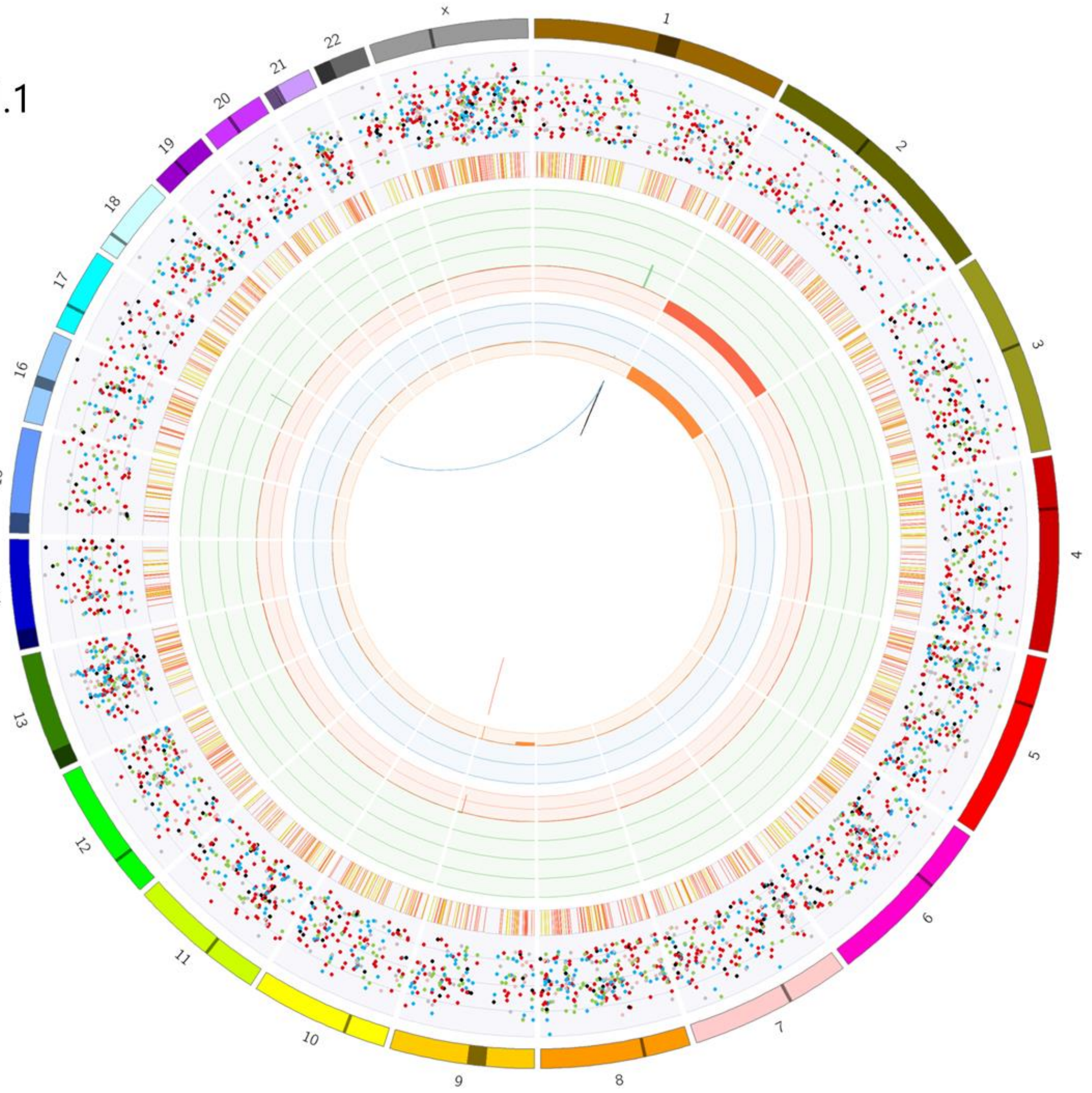

GCPA013T2.1

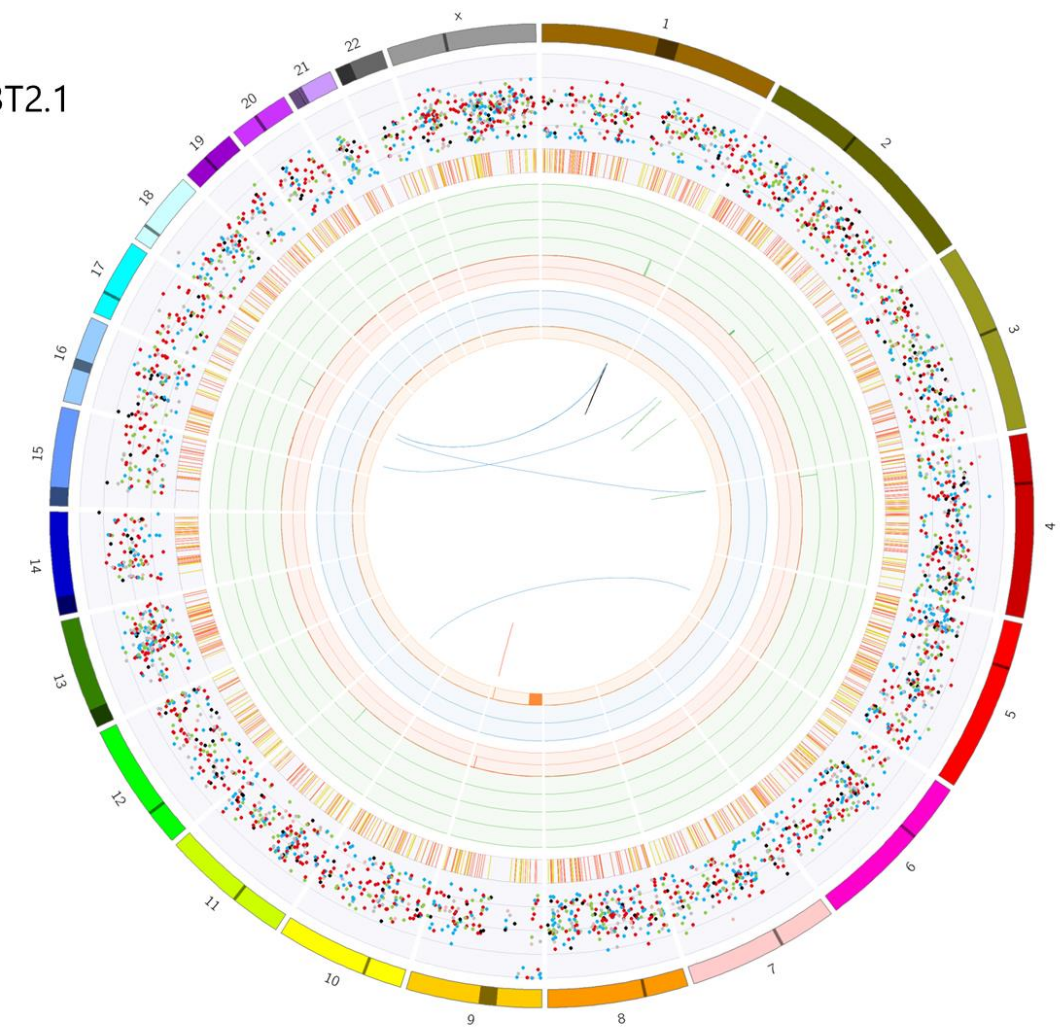

GCPA013T2.2

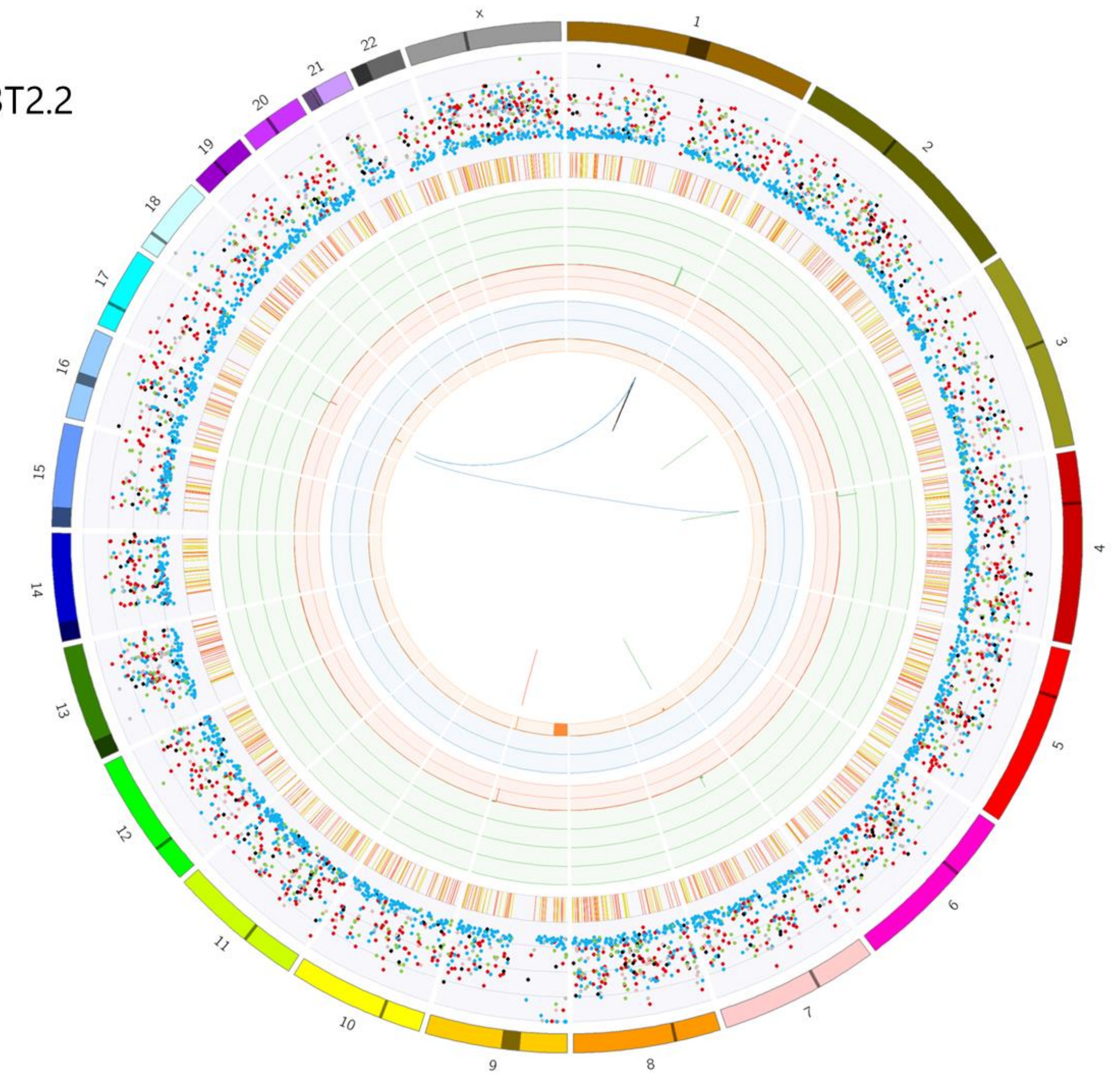

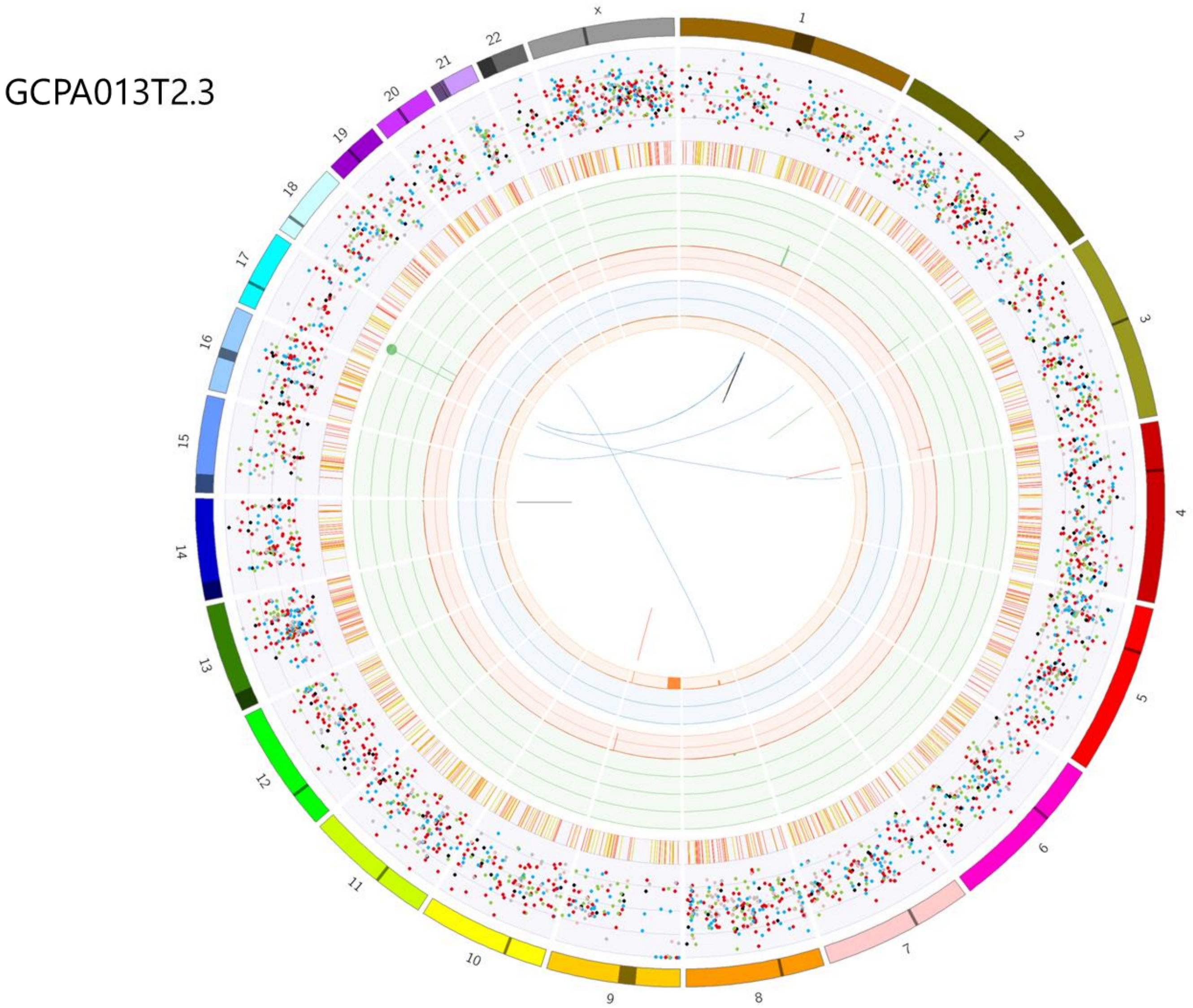
GCPA013T2.3
x
1
2
3
4
5
6
7
8
9
10
11
12
13
14
15
16
17
18
19
20
21
22

GCPA013T2.4

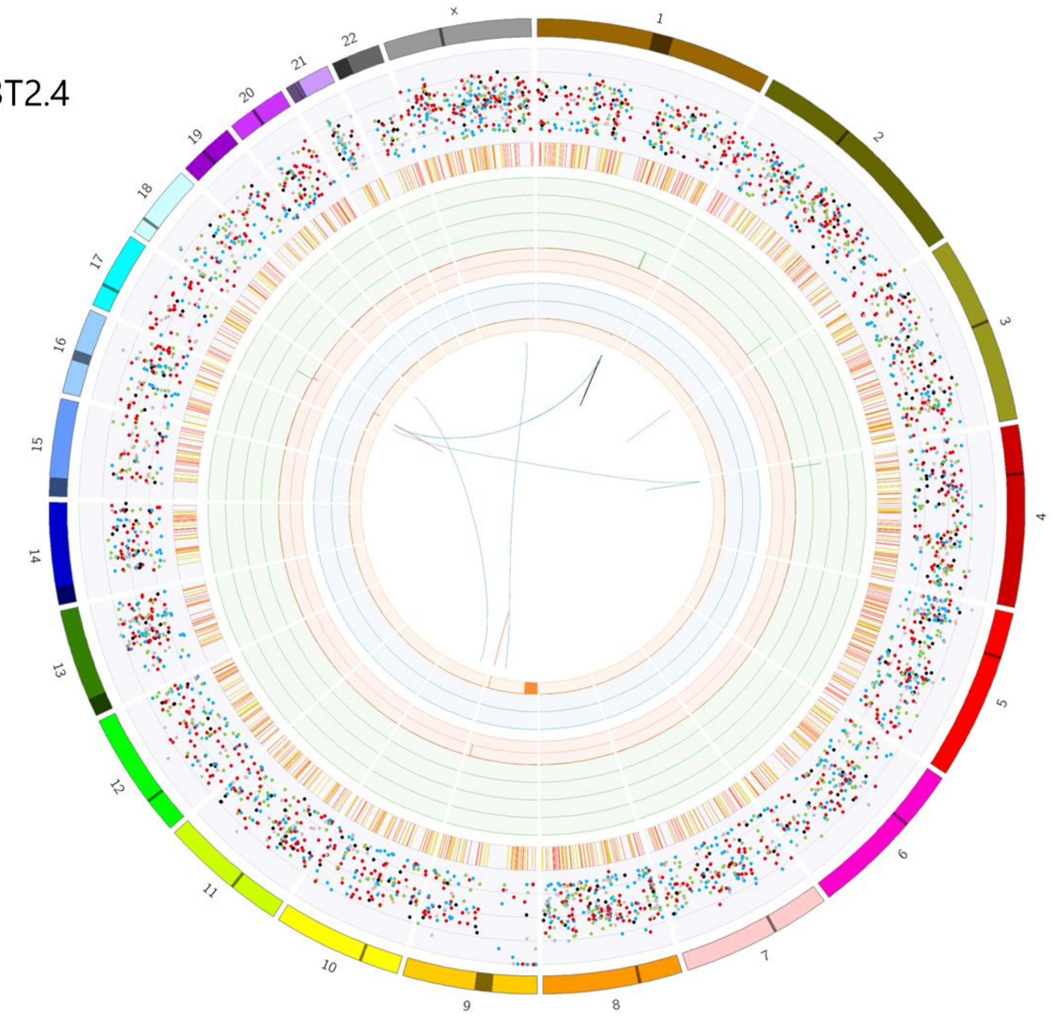

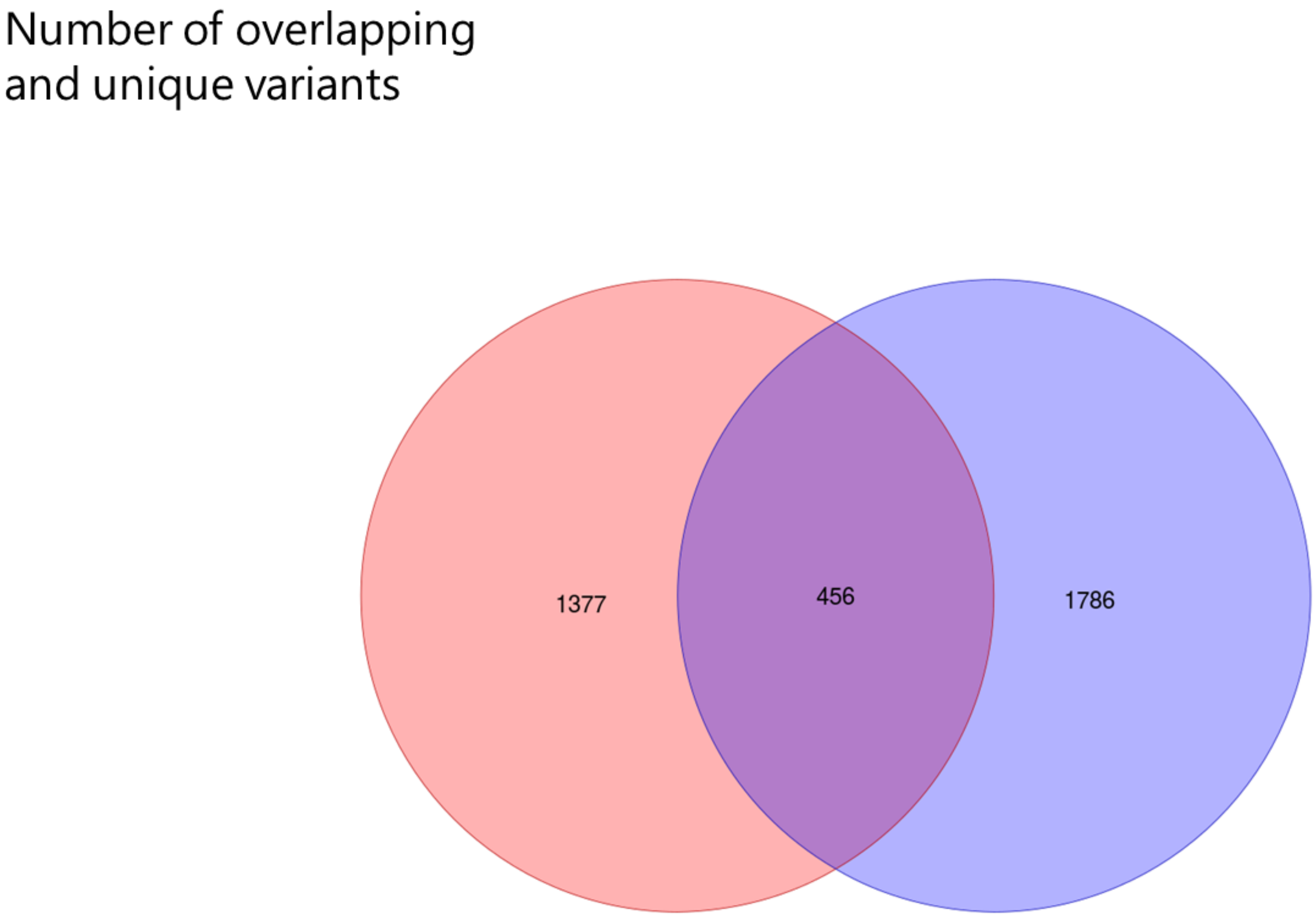
Number of overlapping
and unique variants
1377
456
1786

GCPA022T1.1

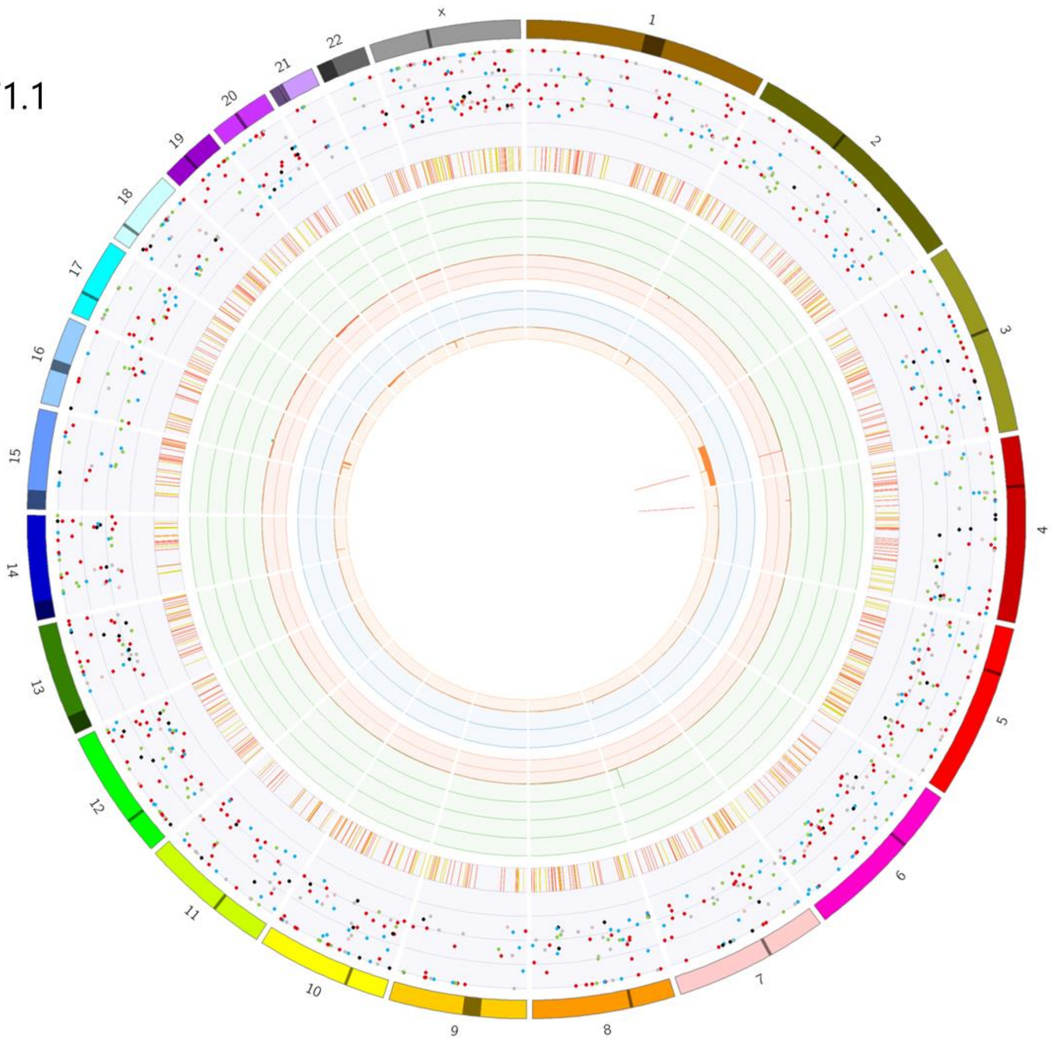

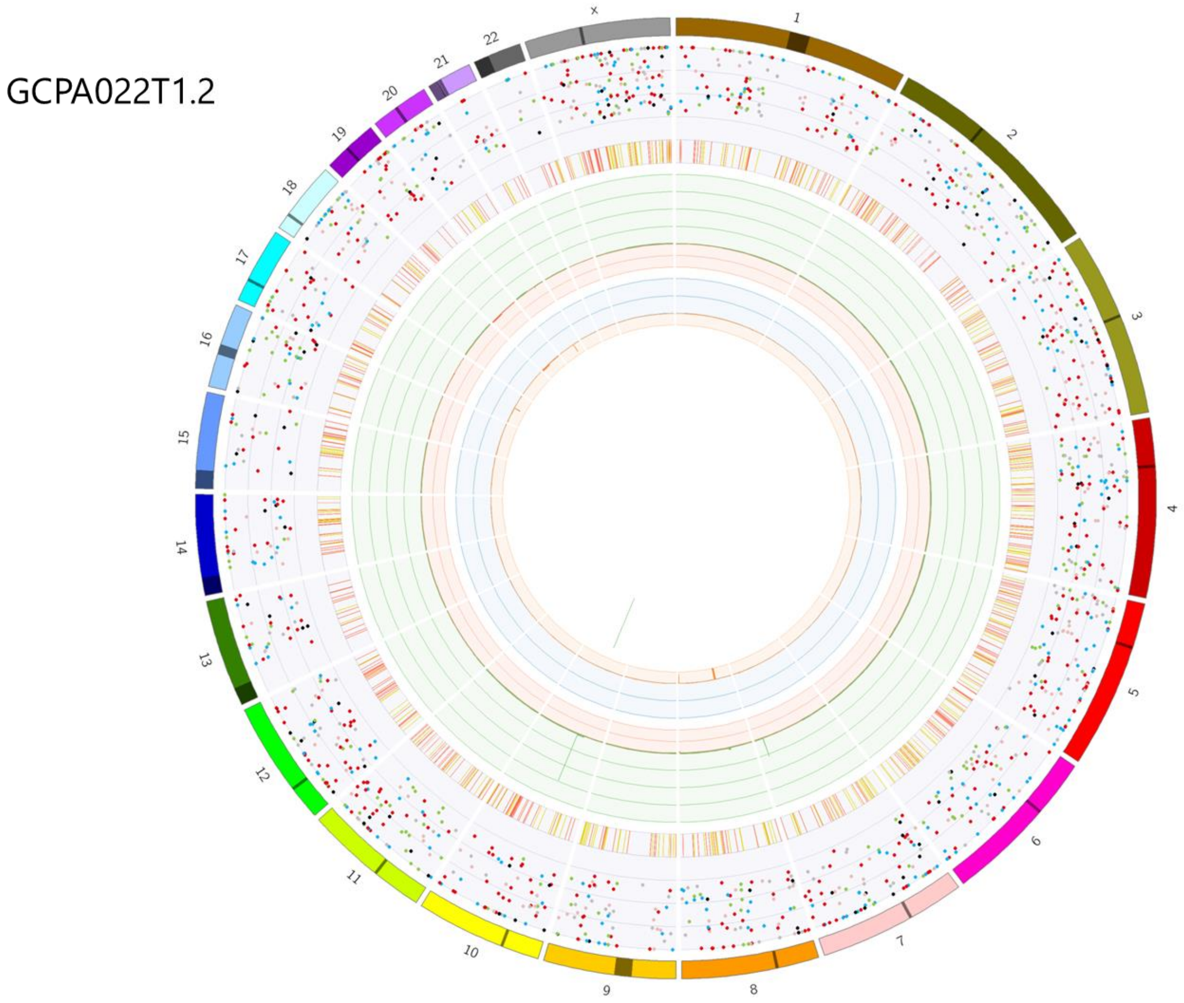
GCPA022T1.2
X
1
2
3
4
5
6
7
8
9
10
11
12
13
14
15
16
17
18
19
20
21
22

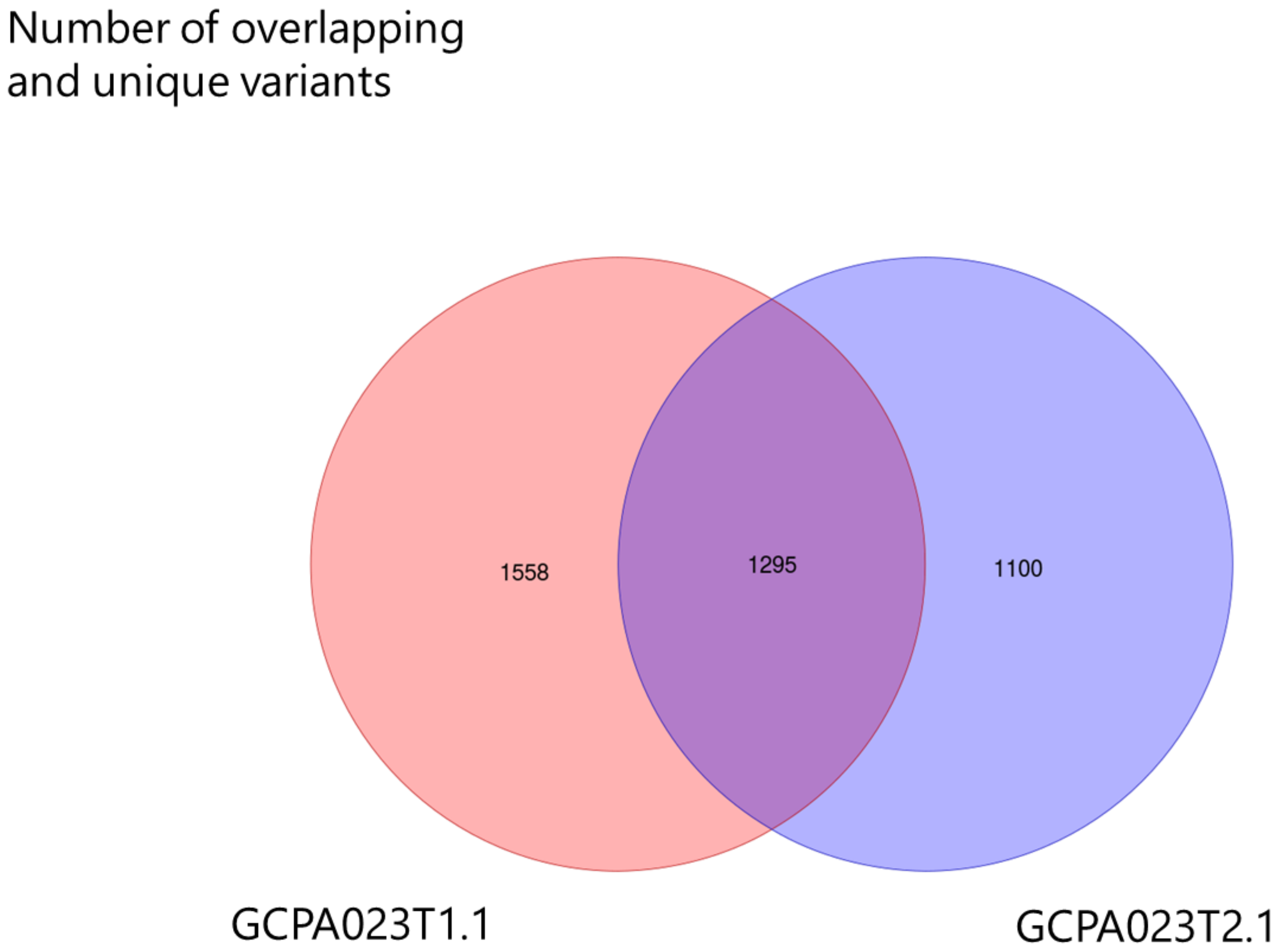
Number of overlapping
and unique variants
1558
1295
1100
GCPA023T1.1
GCPA023T2.1

GCPA023T1.1

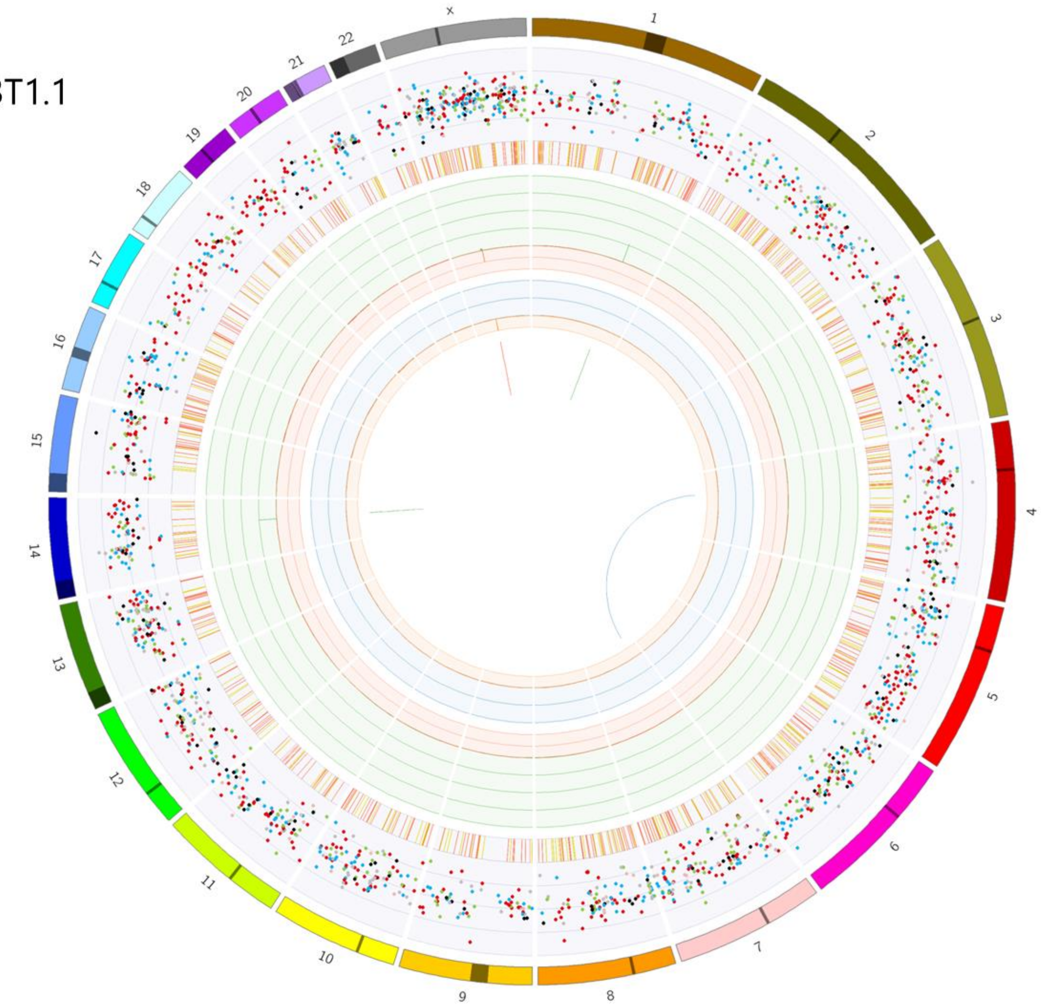

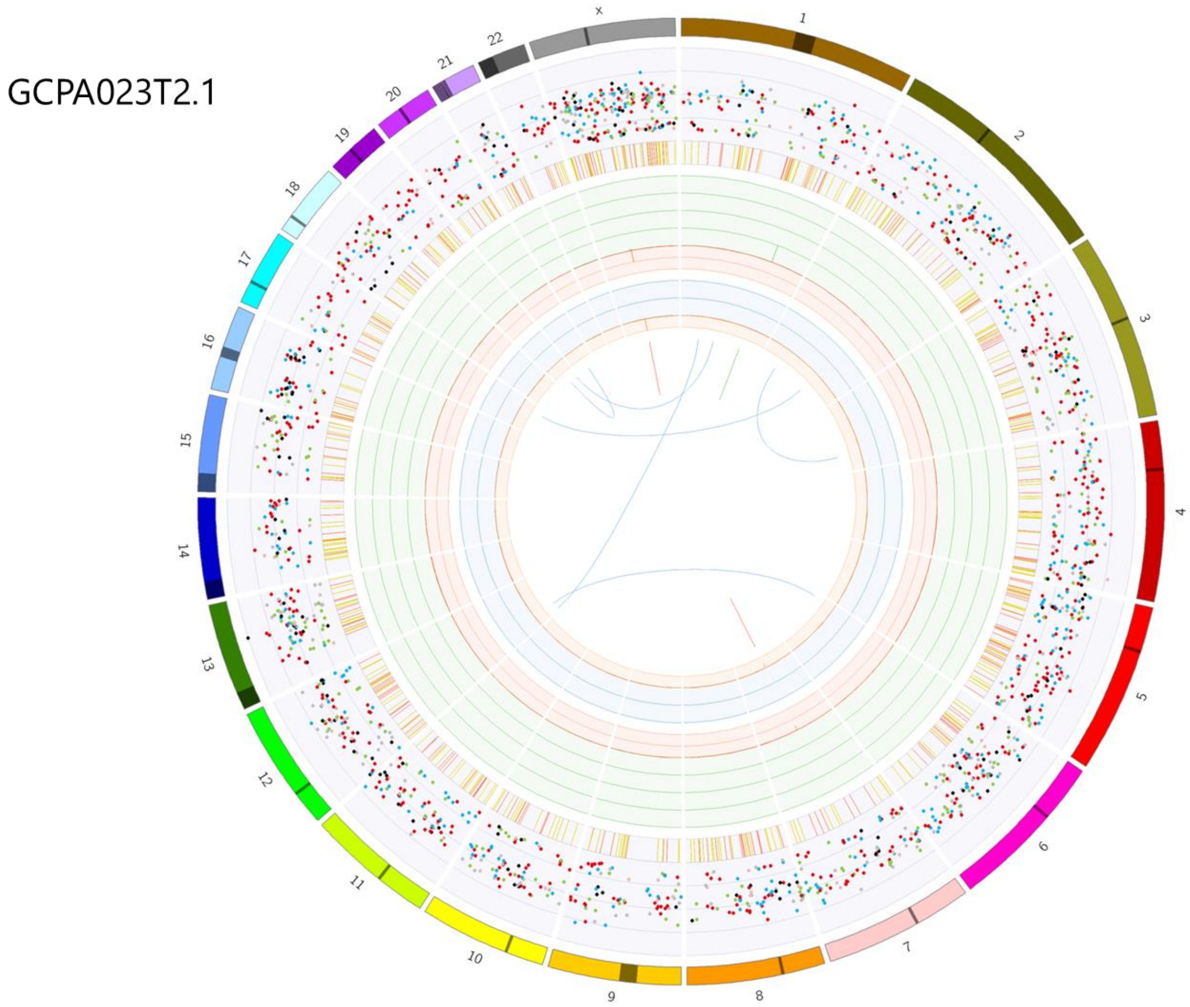

Figure S3b. Circos plots and venn diagrams from all intra-patient comparisons.

Details on CIRCOS plot

- The outer first circle shows the chromosomes. The darker shaded areas represent large gaps in the human reference genome: i.e. regions of centromeres, heterochromatin & missing short arms.
- The second circle shows the somatic variants (incl. exon, intron and intergenic regions). Somatic variants are further divided into an outer ring of single nucleotide polymorphism (SNP) allele frequencies and an inner ring of short insertion/deletion (INDEL) locations. SNP allele frequencies have been corrected for tumor purity and scale from 0 to 100%. Each dot represents a single somatic variant. SNPs are colored according to the type of base change (e.g. C>T/G>A in red) and are in concordance with the coloring used in Alexandrov et al. 2013 Nature paper that describes the use of mutational signatures. INDELs are colored yellow and red for insertions and deletions respectively.
- The third circle shows all observed tumor purity adjusted copy number changes, including both focal and chromosomal somatic events. Copy number losses are indicated in red, green shows regions of copy number gain. The scale ranges from 0 (complete loss) to 6 (high level gains). If the absolute copy number is > 6 it is shown as 6 with a green dot on the diagram.
- The fourth circle represents the observed 'minor allele copy numbers' across the chromosome. The range of the chart is from 0 to 3. The expected normal minor allele copy number is 1, and anything below 1 is shown as a loss (orange) and represents a LOH event. Minor allele copy numbers above 1 (blue) indicate amplification events of both A and B alleles at the indicated locations.
- The innermost circle displays the observed structural variants within or between the chromosomes. Translocations are indicated in blue, deletions in red, insertions in yellow, tandem duplications in green and inversions in black.

© 2020 by the authors. Licensee MDPI, Basel, Switzerland. This article is an open access article distributed under the terms and conditions of the Creative Commons Attribution (CC BY) license (http://creativecommons.org/licenses/by/4.0/).
